# Genome-Scale Reconstruction of the Metabolic Network in *Oenococcus oeni* to Assess Wine Malolactic Fermentation

**DOI:** 10.3389/fmicb.2017.00534

**Published:** 2017-03-30

**Authors:** Sebastián N. Mendoza, Pablo M. Cañón, Ángela Contreras, Magdalena Ribbeck, Eduardo Agosín

**Affiliations:** Laboratory of Biotechnology, Department of Chemical and Bioprocess Engineering, School of Engineering, Pontificia Universidad Católica de ChileSantiago, Chile

**Keywords:** genome-scale metabolic model, malolactic fermentation, lactic acid bacteria, *Oenococcus oeni*, physiological ethanol response

## Abstract

*Oenococcus oeni* is the main responsible agent for malolactic fermentation in wine, an unpredictable and erratic process in winemaking. To address this, we have constructed and exhaustively curated the first genome-scale metabolic model of *Oenococcus oeni*, comprising 660 reactions, 536 metabolites and 454 genes. *In silico* experiments revealed that nutritional requirements are predicted with an accuracy of 93%, while 14 amino acids were found to be essential for the growth of this bacterial species. When the model was applied to determine the non-growth associated maintenance, results showed that *O. oeni* grown at 12% ethanol concentration spent 30 times more ATP to stay alive than in the absence of ethanol. Most of this ATP is employed for extruding protons outside of the cell. A positive relationship was also found between specific consumption rates of fructose, amino acids, oxygen, and malic acid and the specific production rates of erythritol, lactate, and acetate, according to the ethanol content of the medium. The metabolic model reconstructed here represents a unique tool to predict the successful completion of wine malolactic fermentation carried out either by different strains of *Oenococcus oeni*, as well as at any particular physico-chemical composition of wine. It will also allow the development of consortium metabolic models that could be applied to winemaking to simulate and understand the interactions between *O. oeni* and other microorganisms that share this ecological niche.

## Introduction

Malolactic fermentation (MLF) is a key step in the production of most red wines, as well as some white and sparkling wines. This process is primarily responsible for lowering the acidity of wine, and also generates other benefits, such as improving aroma and flavor complexity; as well as increasing the biological stability of the resulting wines (Davis et al., 1985; Henschke, 1993; Bartowsky et al., 2002). This secondary fermentation, mainly carried out by lactic acid bacteria (LAB), involves the NAD^+^ and manganese-dependent decarboxylation of L-malate to L-lactate and CO_2_ (Kunkee, 1974; Williams et al., 1984). Failures in the onset and completion of malolactic fermentation are commonplace worldwide, which inconveniently delays the overall process of winemaking and therefore results in significant economic losses.

*Oenococcus oeni* is the main species involved in MLF due to its ability to grow in harsh environments, such as wine. This bacterial species is characterized by its ability to grow at high ethanol content (>13% v/v), low pH (<3.5), limited nutrient availability and high sulphite concentration (<50 ppm) (Bauer and Dicks, 2004; Bartowsky, 2005; Zapparoli et al., 2009). Consequently, the success of this secondary fermentation depends on the ability of *O. oeni* to cope with these hostile conditions (Gockowiak and Henschke, 2003; Le Marrec et al., 2007). Several studies have been conducted to understand the metabolism of *O. oeni* under oenological culture conditions. Despite these efforts, MLF remains an unpredictable, capricious and precarious operation of the winemaking process. Indeed, its onset and completion can take weeks or even months (Bartowsky et al., 2015).

Genome sequencing has paved the way to a deeper understanding of this microorganism. Mills et al. (2005) reported that the circular chromosome of *O. oeni* strain PSU-1 contained 1,780,517 nucleotides, with a guanine–cytosine (GC) content of 38%. Borneman et al. (2012) found important genomic differences among several O. *oeni* strains through a comparative analysis of the *O. oeni* pan genome, employing *O. oeni* PSU-1 strain as a reference. More recently, Campbell-Sills et al. (2015) reviewed the population structure of many *O. oeni* strains using comparative genomics, and confirmed that the distribution of 50 strains can be divided into two major groups, according to their ecological niche: wine or cider. Transcriptomic and proteomic analyses of *O. oeni* strains cultivated under wine-simulated conditions showed that the environment strongly affects *O. oeni* stress-responses at both levels (Costantini et al., 2015; Olguín et al., 2015). Despite the bioinformatic tools employed for these studies, a full systemic understanding of the metabolic capabilities and behavior of this malolactic bacterium under extreme environments would strongly benefit from the reconstruction of a genome–scale metabolic model able to integrate the current knowledge of this LAB.

Genome annotation, databases and primary literature (Feist et al., 2009), along with specific collection of biochemical reactions and associated genes that describe the cell metabolism of a specific organism, can be employed for the reconstruction of the metabolic network at the genome scale (Thiele and Palsson, 2010). A genome-scale metabolic model (GEM) is a mathematically structured format of different types of biological knowledge that is used to perform computational and quantitative queries to answer questions about the capabilities of an organism and its likely phenotypic states. GEMs have primarily focused on six applications: (1) metabolic engineering, (2) model-driven discovery, (3) prediction of cellular phenotypes, (4) analysis of biological network properties, (5) studies of evolutionary processes, and (6) models of interspecies interactions (McCloskey et al., 2013). Initially, these models only considered well -characterized organisms; nevertheless, the interest in the generation of metabolic models of less characterized and complex biological systems has progressively increased, including the GEMs of several lactic acid bacteria, such as *Lactococcus lactis* (Oliveira et al., 2005; Oddone et al., 2009; Verouden et al., 2009; Flahaut et al., 2013), *Lactobacillus plantarum* (Teusink et al., 2006) and *Streptococcus thermophilus* (Pastink et al., 2009).

In this work, we constructed the first genome-scale metabolic model of an *O. oeni* strain (named iSM454 model) to provide a tool for simulating the metabolism, nutritional requirements, and specific growth rate of this microorganism under the harsh conditions of winemaking. Here we report the general features of the model, as well as its prediction performance. The resulting metabolic model was employed to assess the metabolic capabilities, limitations and potential of this LAB to successfully accomplish malolactic fermentation in wine.

## Materials and methods

### Construction of the GEM

**Figure 1:**
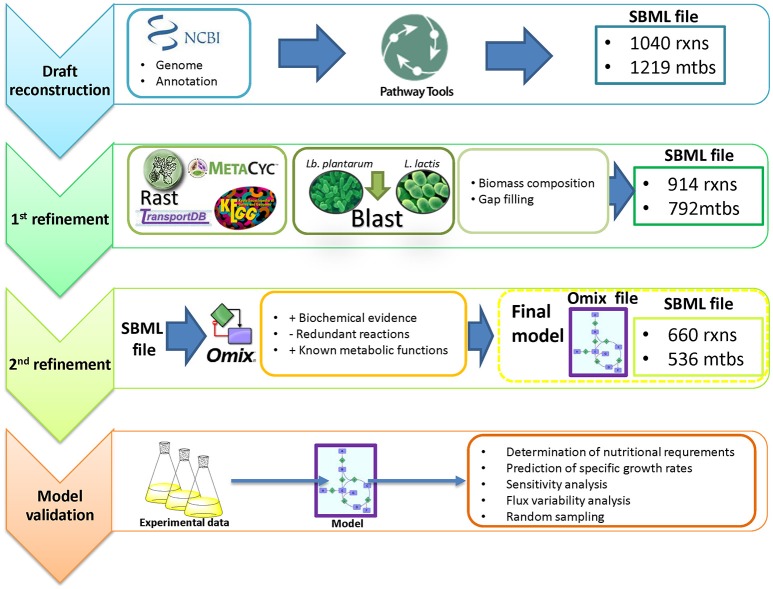
**Genome-scale reconstruction of the metabolic network in *Oenococcus oeni* PSU-1, and model validation**.

The model was constructed following the protocol described by Thiele and Palsson (2010) (Figure 1). As a starting point, we generated a draft reconstruction with Pathway Tools™ version 16.5 (Karp et al., 2002) from the NCBI reference genomic sequence NC_008528.1 of *O. oeni* PSU-1. The model was then manually curated consulting scientific literature and the online databases KEGG™^1^ (Kyoto Encyclopedia of Genes and Genomes, Kanehisa, 2000), MetaCyc™^2^ (Caspi et al., 2014) and TransportDB™^3^ (Membrane Transport Database, Ren et al., 2007). Comparison with other genome annotations such as RAST^4^, as well as with previous models MG1363 (Flahaut et al., 2013) and WCFS1 (Teusink et al., 2006) from *Lactococcus lactis* and *Lactobacillus plantarum* respectively, was conducted in order to find missing reactions (Table 1). The presence of the enzyme(s) responsible for carrying out these reactions in the genome were subsequently checked using the online available version of Basic Local Alignment Tools^5^ (BLAST™, Madden, 2002).

**Table 1:** **Comparison between GEM of *Oenococcus oeni* PSU-1, *Lactobacillus plantarum* WCFS1 and *Lactococcus lactis* IL1403**.

	**Common reactions with**			**Unique reactions**	**Total reactions**
	***Lb. plantarum***	***Lc. lactis***	**both models**	**in *O. oeni***	**in *O. oeni***
Amino acids metabolism	39 (21)	40 (20)	32	13	60
ATP maintenance	1 (0)	1 (0)	1	0	1
Beta-oxidation	0 (8)	3 (5)	0	5	8
Biomass assembly	0 (1)	0 (1)	0	1	1
Carbohydrates metabolism	49 (26)	43 (32)	34	17	75
Citrate degradation	5 (2)	7 (0)	5	0	7
Exchange reactions	66 (44)	55 (55)	44	33	110
EPS biosynthesis	5 (5)	4 (6)	4	5	10
Fatty acid biosynthesis	40 (23)	4 (59)	2	21	63
Glycolipids metabolism	10 (9)	13 (6)	9	5	19
Glutathione redox reactions	1 (3)	1 (3)	1	3	4
Inorganic metabolism	5 (7)	2 (10)	1	6	12
Macromolecules assembly	2 (4)	4 (2)	2	2	6
Malolactic fermentation	1 (0)	1 (0)	1	0	1
Menaquinol metabolism	2 (3)	1 (4)	1	3	5
Nucleotides metabolism	53 (19)	53 (19)	47	13	72
Peptidoglycan biosynthesis	9 (4)	10 (3)	7	1	13
Terpenes biosynthesis	9 (6)	11 (4)	9	4	15
Thioredoxin redox reactions	0 (1)	0 (1)	0	1	1
Transport	30 (101)	24 (107)	15	92	131
Ubiquinol metabolism	0 (3)	0 (3)	0	3	3
Vitamins metabolism	27 (16)	20 (23)	15	11	43
TOTAL	354 (306)	297 (363)	230	239	660

*The table shows common reactions between models, unique reactions, and total reactions in the model of O. oeni. Figures in parentheses indicate unique reactions in the model respect to Lb. plantarum or Lc. lactis models*.

For proper network visualization, the model was then manually exported into Omix™ version 1.8 (Droste et al., 2011). Then, it was exported to MATLAB™ version 2013b, as an SBML file, and curated using Cobra Toolbox version 2.0. Flux Balance Analysis (FBA), Flux Variability Analysis (FVA), single gene deletion and single reaction deletion were performed to explore the metabolic capabilities of the network.

### Mathematical formulation

FBA is a widely used approach for studying biochemical networks. Among its many uses, FBA has been applied for predicting gene essentiality, quantifying the cellular growth under cultivation conditions and identifying by-product secretion (Park et al., 2009). This approach allows us to calculate the flow of metabolites through the network (Orth et al., 2010). Specifically, FBA quantifies the flux distribution by linear programming (LP) on the basis of stoichiometry of metabolic reactions and mass balances around metabolites under the pseudo-steady state, or stationary assumption (Park et al., 2009).

We employed FBA to calculate the optimal distribution of metabolic fluxes of an underdetermined system of stoichiometric equations (Orth et al., 2010). Following formal procedures, the GEM iSM454 was represented by a stoichiometric matrix *S*, in which the row *i* represents the *i*th reaction and the column *j* the *j*th metabolite of the network. Under a pseudo-steady state assumption, the concentration of metabolites was considered to be constant, which is stated by the equation *S* × *v* = *0*, where *v* is the vector of reaction fluxes. To determine the flux distribution, biomass formation was defined as the objective function and optimized through LP (Equation 1). Gurobi 6.5^6^ (Gurobi Optimization Inc, 2016) was chosen as the optimization solver.

(1) $$\begin{array}{r} \;Max\;\mu \end{array}$$

$$\begin{array}{r} Subjet\;to\;S\times v=0\\ v_{l}\leq v_{i}\leq v_{u}\;\forall \;i=1\;\ldots n \end{array}$$

Where μ is the specific growth rate [1 h^−1^], *v*_*i*_ is the flux through reaction *i*, *v*_*l*_, and *v*_*u*_ are the lower and upper bounds for that reaction, and n is the number of reactions of the reconstruction.

### Network evaluation

#### Determination of nutritional requirements

We ran *in silico* experiments to determine the nutritional requirements of the *O. oeni* PSU-1 strain and then we compared the results with experimental data to validate iSM454. For this purpose, we used the 44 single omission experiments described by Terrade and Mira de Orduña (2009) and the 17 experiments for alternative carbon sources described by Beelman et al. (1977). Specific restrictions were set to simulate each medium (Supplementary Data Sheet 1). For each medium, we defined the restrictions required to simulate the nutrients included in the medium by allowing flux only through exchange reactions corresponding to those nutrients. Otherwise, lower and upper bounds of exchange reactions representing substrate uptake were set to zero.

For single omission experiments, each of the 44 nutrients was removed, one by one, and an optimization run was carried out each time. Nutrients that inhibited growth when removed were considered to be essential. We considered that growth was inhibited when the specific growth rate of the auxotrophic mutant was less than 20% that of the wild type. For the second set of experiments, the 17 alternative carbon sources were tested independently by performing an optimization run in each case. Carbon sources that allowed growth without the presence of other carbon sources were considered to sustain growth by themselves.

For both sets of experiments, results were classified as true positives (growth observed both *in vivo* and *in silico*), true negatives (no growth observed neither *in vivo* nor *in silico*), false positives (growth *in silico* but not *in vivo*) and false negatives (growth *in vivo* but not *in silico*).

From these predictions, we calculated critical statistical parameters that define model performance, i.e., sensitivity, specificity, precision, negative predictive value, accuracy, and the F-score, as follows:

(2) $$\begin{array}{r} Sensitivity=TP/\left(TP+FN\right) \end{array}$$

(3) $$\begin{array}{r} Specificity=TN/\left(TN+FP\right) \end{array}$$

(4) $$\begin{array}{r} Precision\;\left(PPV\right)=TP/\left(TP+FP\right) \end{array}$$

(5) $$\begin{array}{r} Negative\;predicted\;value\;\left(NPV\right)=TN/\left(TN+FN\right) \end{array}$$

(6) $$\begin{array}{r} Accuracy=\left(TP+TN\right)/\left(TP+TN+FP+FN\right) \end{array}$$

(7) $$\begin{array}{r} F-score=2\left(precision\;\times \;sensitivity\right)/\left(precision\;+\;sensitivity\right) \end{array}$$

#### Prediction of ATP-maintenance

Following standard procedures, we added an equation (Equation 7) to represent non-growth associated maintenance (NGAM).

(8) $$\begin{array}{r} ATP+H_{2}O\to ADP+P_{i}+H^{+} \end{array}$$

The values of NGAM were determined from the model for each experimental condition. For this purpose, we first fixed the consumption and production rates of different metabolites and then we progressively increased the NGAM from 0 to 5 mmol gDW^−1^ h^−1^. At each iteration, the growth rate was maximized and the error between the experimental and predicted growth rate was calculated. The value that minimized the error between the experimental and predicted growth rate was chosen as the ATP required for maintenance of cellular processes.

The experimental rates included in the model were specific consumption rates of glucose, fructose, citrate, L-malate, L-cysteine, L-serine, L-threonine; it also included specific production rates of D-mannitol, L-lactate, D-lactate, acetate, erythritol, and ethanol. They were calculated from experimental data of two batch cultures containing 0 and 12% ethanol, respectively, run in duplicate (see below).

#### Experimental determination of specific growth rates and consumption/production rates

An *O. oeni* PSU-1 preculture was prepared from a frozen stock by inoculating 100-ml Erlenmeyer flasks containing 75 ml MRS (Man, Rogosa, and Sharpe) medium (De Man et al., 1960), supplemented with 0.5 g L^−1^ of cysteine. Before inoculation, the cells were subjected to ethanol adaptation. For this purpose, we serially passaged every culture, starting from 1% ethanol (v/v) to reach 0 or 12% ethanol concentration (v/v) in each culture.

The adapted cells were inoculated in 50 mL flasks containing 35 mL of a chemically defined, wine-like, culture medium to achieve an initial optical density at 600 nm (OD600) of approximately 0.2. We employed the modified culture medium described by Terrade and Mira de Orduña (2009), at an initial pH adjusted to 4.8.

The flasks were incubated at 25°C, without stirring. OD600 was periodically measured to calculate the specific growth rate. At the same time, the content from each flask was centrifuged, the supernatant was collected and an aliquot was injected in a Lachrom L-700 HPLC system (Hitachi, Japan) equipped with a Diode Array and a Refractive Index detectors (Merck Hitachi, Japan). Organic acids, alcohols and sugars were separated using an Aminex HPX-87H ion exchange carbohydrate-organic acid column (Bio-Rad, USA) and quantified, as described previously (Varela et al., 2003).

#### Amino acids essentiality assay

An *O. oeni* PSU-1 preculture was prepared from a frozen stock as described above. The cells (not pre-adapted in ethanol) were inoculated in 50 mL flasks containing 35 mL of the same chemically defined, wine-like, culture medium, but lacking the amino acids evaluated for essentiality (glutamate, glutamine, asparagine, and threonine), one amino acid per flask, in duplicate. The flasks were incubated at 25°C during 13 days, without stirring, and OD600 was periodically measured to calculate the specific growth rate.

#### Sensitivity analysis

For each optimization using experimental data, non-zero reduced costs were extracted from the solver solution and employed for quantifying the impact of changing a capacity constraint on the objective flux. Scaled reduced costs were calculated as follows:

(9) $$\begin{array}{r} W_{i}\;=\;w_{i}\;\times \;q_{i}/\mu \end{array}$$

Where *w*_*i*_ represents the reduced cost, *q*_i_ the flux through exchange reaction *i*, and μ the specific growth rate. *W*_*i*_, the scaled reduced cost of the exchange reaction *i*, represents the fractional change in biomass obtained by a fractional variation in compound *i*. Reactions that showed both, a non-zero reduced cost and a non-zero scaled reduced cost, were further analyzed.

#### Flux variability analysis

FVA was carried out by minimizing and maximizing the flux through each reaction, under either unconstrained or constrained conditions. Span range was sorted by magnitude and plotted.

Reactions unable to carry flux were considered blocked. For each of these, the cause of the obstruction was investigated by finding dead-end metabolites; these were determined searching for those metabolites that were only consumed or produced in the stoichiometric matrix. Additionally, we determined dead-ends by adding a demand (maximizing the flux) or a sink (minimizing the flux) reaction for each metabolite. Metabolites were considered dead-ends if the model was unable to produce, nor consume them.

#### Random sampling

We conducted a random sampling analysis using optGpSampler (Megchelenbrink et al., 2014), an efficient algorithm based on the Monte Carlo procedure *hit and run* (Smith, 1984). For each experimental condition, we set the algorithm parameters in order to sample 100.000 points using 500 steps between each point.

We applied the algorithm to explore the solutions at an optimal specific growth rate for each experimental condition. For this purpose, the model was restricted with the calculated optimal growth rate and the corresponding experimental consumption/production rates. Then, we applied the algorithm for determining the 100,000 flux distributions that accomplished these restrictions. For every condition, we found the 50 reactions that showed the greatest flux variations among the distributions. We classified these reactions according to pathways and then we sorted pathway frequency.

We also applied this algorithm to explore the solutions near the optimal specific growth rate, by following the same procedure described above. For this purpose, the lower bound for specific growth rate was fixed at 90% of the optimal, and the upper bound, at the optimal specific growth rate.

## Results

### General features of the GEM of *Oenococcus oeni* PSU-1 strain

The iSM454 model (Supplementary Data Sheet 2 and Supplementary Figure 1) consists of 660 reactions, 536 metabolites and 454 genes. 24% of the 1864 genes described in the genome annotation (Makarova et al., 2006) were included in the model. 68% of the reactions are associated at least to one gene. The model includes 132 transport reactions, 110 exchange reactions, 3 extracellular reactions (dextran synthesis, heteropolysaccharide synthesis and cellulose degradation) and 411 intracellular reactions. It contains 148 blocked reactions, i.e., reactions that do not carry flux, including 107 dead-ends (Table 2).

**Table 2:** **Main features of genome scale metabolic model of *Oenococcus oeni* PSU-1**.

Total Genes	1864
Included genes	454
Total Pathways	91
Total Reactions	660 (448)^a^
Intracellular	413 (340)^a^
Extracellular	3 (2)^a^
Transport	133 (106)^a^
Exchange	111
Spontaneous	8
Assembly	7
Non-genes associated^b^	101
Blocked	148
With dead-ends	107
Without dead-ends^c^	41
Total Metabolites	536
Intracellular	434
Extracellular	102

a *In brackets, number of reactions associated to genes*.

b *Exchange reactions are not considered*.

c *Without dead-ends, but associated to reactions with dead-ends*.

Connectivity corresponds to the number of reactions where a metabolite participates. As shown in Table 3, the iSM454 model presents a similar connectivity, in relation to key metabolites, with WCFS1 (*Lb. plantarum*), IL1403 (*Lc. Lactis*), and Yeast 7 (*S. cerevisiae*) models (Oliveira et al., 2005; Teusink et al., 2006; Aung et al., 2013). The connectivity analysis (Supplementary Figure 2) indicates that 240 out of the 536 metabolites included in the iSM454 model participate in two metabolic reactions: 100 in three reactions, and only 40 in more than seven reactions.

**Table 3:** **Comparison of key metabolites connectivity between iSM454 model (*O. oeni*), WCFS1 (*Lb. plantarum*), IL1403 (*Lc. lactis*) and Yeast 7 (*S. cerevisiae*) models**.

	**Connectivity**			
	***O. oeni* iSM454**	***Lb. plantarum* WCFS1**	***Lc. lactis* IL1403**	***S. cerevisiae* Yeast 7**
NADPH	32	34	37	58
NADP^+^	33	35	39	58
NADH	39	57	36	36
PPi	43	63	50	69
NAD^+^	43	62	40	42
Pi	96	93	101	155
ADP	111	113	113	121
ATP	128	148	130	158
H_2_O	162	165	141	269
H^+^	230	382	110	433

### Metabolic refinement of the iSM454 model

The metabolism of carbohydrates, amino acids, and fatty acids, as well as macromolecular assembly, transport, and ATP production, were thoroughly checked at this stage, as described below.

#### Carbohydrates metabolism

*Oenococcus oeni* is a heterofermentative bacterium. It consumes hexoses through the 6-phospho-gluconate pathway and produces carbon dioxide, D-lactate, acetate, and/or ethanol. The main metabolized hexoses are glucose and fructose. The latter can also be transformed into mannitol or erythritol to fulfill the demand for NAD^+^ required in the heterolactic fermentative pathway. Even though the genes related to mannitol and erythritol biosynthetic pathways were not found in the PSU-1 genome, these pathways were included in the model to account for reported experimental data (Beelman et al., 1977). The membrane transporters of these and other carbohydrates—arabinose, ribose, melibiose, mannose, fucose, xylose, and galactose—were found using PathoLogic (Dale et al., 2010), which is provided by Pathway Tools, and included in iSM454.

Meanwhile, as *O. oeni* synthesizes exopolysaccharides (EPS) (Ciezack et al., 2010; Dimopoulou et al., 2012, 2014), we included 7 reactions responsible for EPS biosynthesis, associated with 22 genes annotated in the genome.

Finally, we also curated the pathways related to peptidoglycan biosynthesis. The draft reconstruction contained three alternative pathways to synthesize peptidoglycan (pathways I, III, or V). We only left pathway I in the model because it was the most complete, i.e., 9 out of the 10 reactions of this pathway were associated with genes.

#### Amino acids metabolism

The iSM454 model contains the whole biosynthetic pathways for 6 amino acids (alanine, aspartate, glutamine, lysine, proline, and glycine), as arisen from genome annotation (Mills et al., 2005). In particular, the gene dapX encoding for the enzyme diaminopimelate epimerase was absent in the genome annotation and therefore, was added to iSM454 in order to complete lysine biosynthesis pathway (Rodionov et al., 2003). The biosynthetic pathways of the remaining 14 amino acids are incomplete (arginine, asparagine, cysteine, glutamate, histidine, isoleucine, leucine, methionine, phenylalanine, serine, threonine, tryptophan, tyrosine, and valine), in accordance with genomic analysis (Mills et al., 2005).

#### Fatty acids metabolism

*Oenococcus oeni* does not store triglycerides as an energy reserve. Instead, fatty acids are mainly utilized for the construction of the cytoplasmic membrane. The lipid fraction of *O. oeni* is mainly composed by saturated fatty acids (laurate, myristate, palmitate, stearate), unsaturated fatty acids (palmitoleate, oleate, cis-vaccenate) and cyclopropane fatty acids (lactobacillate and dihydrosterculate) (Tracey and Britz, 1989b; Lonvaud-Funel and Desens, 1990; Garbay et al., 1995; Guerrini et al., 2002). Biosynthesis of saturated fatty acids was automatically included into the model by Pathway Tools. Meanwhile, the biosyntheses of unsaturated and cyclopropane fatty acids were manually added to the model with their respective gene associations.

The synthetic routes for cardiolipin, 3-D-glucosyl-1,2-diacylglycerol, L-1-phosphatidyl-glycerol, and lysophosphatidylglycerol, starting from dihydroxyacetone phosphate, were also included.

In relation to β-oxidation of fatty acids, Pathway Tools assigned two genes, *OEOE_1366* and *OEOE_1263*, to the same acyl-CoA synthetase (EC number 6.2.1.3) associated with a generic acyl fatty acid. We therefore manually included these genes and reactions for the canonical catabolism of the above-mentioned saturated fatty acids.

#### Assembly of macromolecules

A modified version of the reported biomass equation of *L. lactis* (Oliveira et al., 2005) was included in iSM454, according to some unique features reported in the literature for *O. oeni*. For example, deoxyribonucleotide content was taken from the genomic analysis for *O. oeni* PSU-1 (Makarova et al., 2006). Fatty acid composition was determined as the average of each individual molecule (Tracey and Britz, 1989b; Lonvaud-Funel and Desens, 1990; Garbay et al., 1995; Guerrini et al., 2002). Amino acids, ribonucleotides and lipids composition correspond to *L. lactis* biomass composition (Oliveira et al., 2005). Similarly, macromolecular elements (proteins, lipids, DNA, and RNA) were included (Oliveira et al., 2005). Finally, the lipoteichoic acid (LTA) synthetic pathway present in the *L. lactis* genome was eliminated because the genes for its synthesis were absent in the *O. oeni's* genome and its presence has not been described in this microorganism (Ribéreau-Gayon et al., 2006).

#### Energy

**Figure 2:**
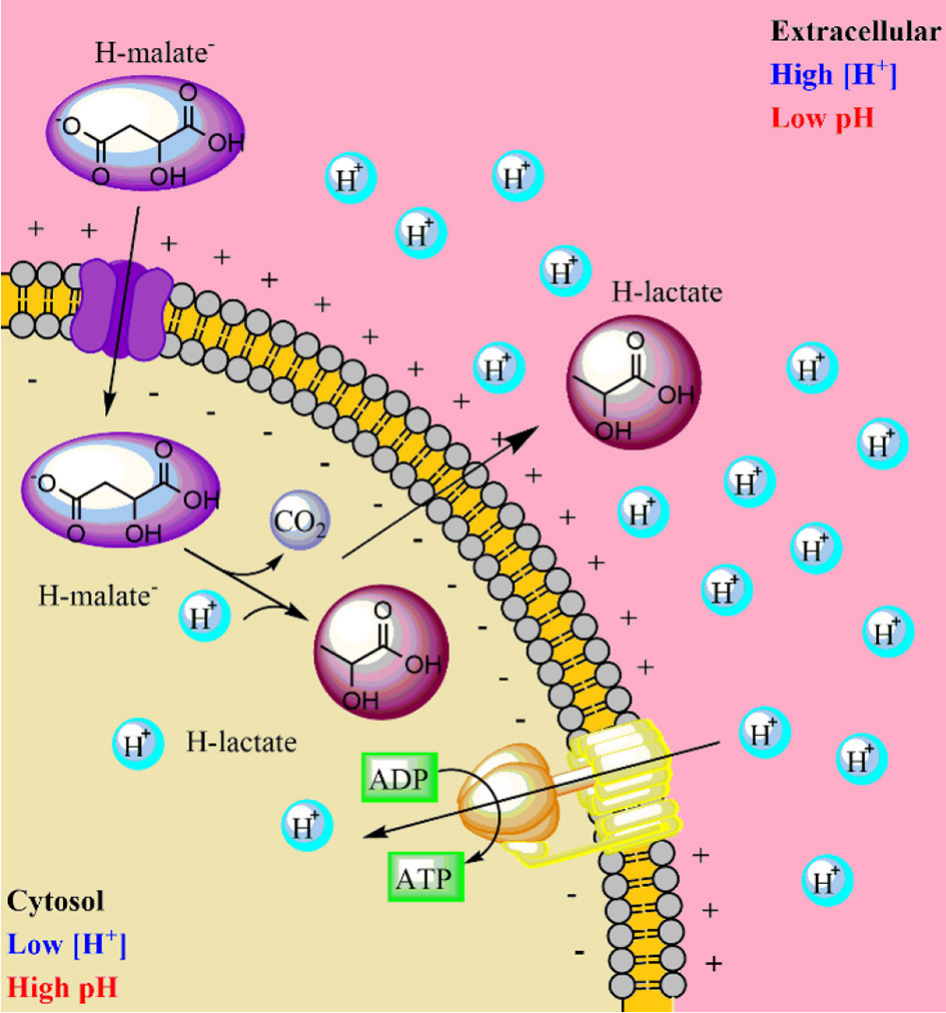
**Electrochemical gradient formation in *Oenococcus oeni* toward the malolactic fermentation**.

Malate metabolism was added to the model, considering the transformation of malic acid to lactic acid by the malolactic enzyme (malate decarboxylase), codified by the *OEOE_1564* gene. In this reaction, a cytosolic proton is consumed, and lactic acid diffuses outside of the cell (Figure 2) (Salema et al., 1996; Konings et al., 1997). The net result of this process is a decrease in the concentration of intracellular protons, contributing to the formation of an electrochemical gradient. Additionally, the citrate lyase complex was lumped into one reaction, directly allowing the conversion of citrate to oxaloacetate. A stoichiometric equation to account for the diffusion of citrate inside *O. oeni* was also added. The model also contains a functional ATP synthase system.

### Model validation

With the aim of determining the functionality of the model, we contrasted the results predicted by iSM454 with experimental data.

#### Determination of *in vivo* amino acids requirements

**Figure 3:**
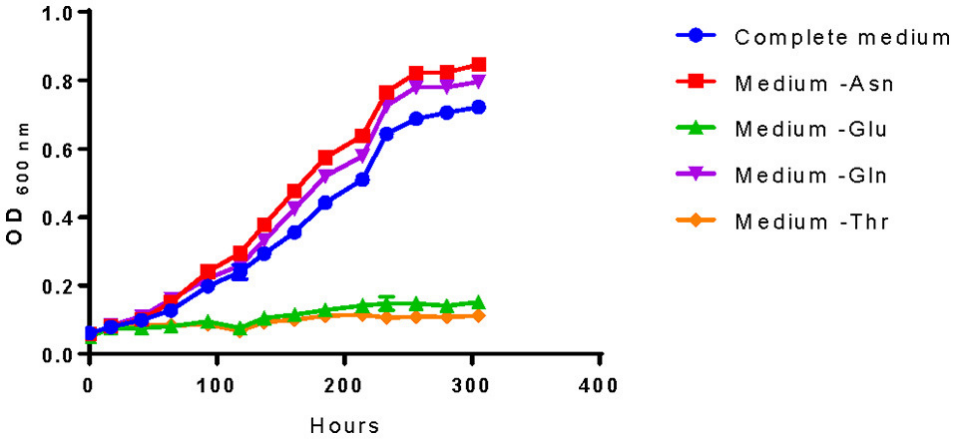
**Amino acids essentiality analysis of *O. oeni* PSU-1**. The amino acids studied are those with controversy with literature. Bacterial growth was compared with the complete medium (and the medium lacking asparagine (- Asn), glutamate (- Glu), glutamine (- Gln), and threonine (- Thr).

Model outputs of the essentiality of some amino acids differed from literature data (Garvie, 1967; Tracey and Britz, 1989a; Fourcassie et al., 1992; Mills et al., 2005; Terrade and Mira de Orduña, 2009). Therefore, we addressed these differences by experimentally evaluating their role on cell growth (Figure 3).

For example, Mills et al. (2005) reported that the genes related to the threonine biosynthetic pathway were all present in PSU-1. However, we identified a pseudogene within this pathway and experimentally demonstrated its essentiality for *O. oeni* PSU-1. On the contrary, even though several genes of the asparagine biosynthetic pathway were not found in the genomic sequence, our experimental results confirmed that *O. oeni* PSU-1 could synthesize this amino acid (Figure 3); and the whole pathway was included in the reconstructed model. In the case of glutamine, both our experimental results and model reconstruction confirmed that this amino acid is not essential, at least for this strain. Finally, *O. oeni* PSU-1 showed auxotrophy for glutamate, in agreement with previous results (Garvie, 1967; Tracey and Britz, 1989a; Fourcassie et al., 1992; Mills et al., 2005; Terrade and Mira de Orduña, 2009).

#### Determination of *in silico* nutritional requirements

**Figure 4:**
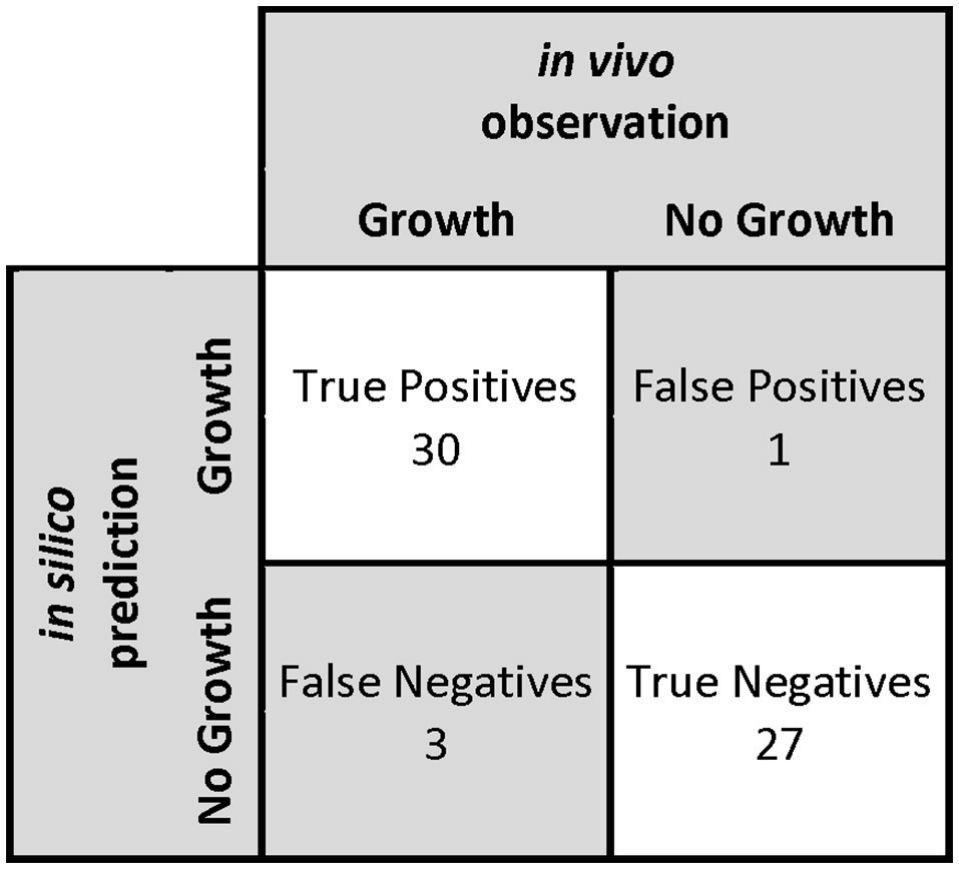
**Confusion matrix used to measure the performance of predictions in the determination of *in silico* nutritional requirements**. The statistical parameters that define model performance are: sensibility of 91%, specificity of 96%, precision of 97%, negative predictive value of 90%, accuracy of 93%, and F-score of 94%.

First, we carried out an *in silico* single omission experiment to compare the nutritional requirements predicted by the model with the experimental data obtained after growth of *O. oeni* in the culture medium of Terrade and Mira de Orduña (2009). Second, we tested alternative and independent carbon sources to evaluate growth and then we compared these results with the ones obtained by Beelman et al. (1977) (Table 4). Additionally, a confusion matrix was constructed to measure the performance of our predictions (Figure 4). This approach has been used before to assess the GEM quality of *S. cerevisiae* iIN800 (Nookaew et al., 2008) and iLL672 (Kuepfer et al., 2005), as well as for *L. plantarum* (Teusink et al., 2006) and *Y. lypolitica* (Loira et al., 2012).

**Table 4:** **Experimental validation of the iSM454 metabolic model**.

**Nutrient**	***In vivo***	***In silico***	**Result**	**References**	**Nutrient**	***In vivo***	***In silico***	**Result**	**References**
**Carbon sources**					**Amino acids**				
D-Glucose	+	+	TP	1	L-Alanine	+	+	TP	2
Fructose	+	+	TP	1	L-Arginine	−	−	TN	2,3,4,5
D-Ribose	−	−	TN	2	L-Asparagine	+	+	TP	2
Threhalose	+	+	TP	1	L-Aspartic acid	+	+	TP	2,3,4,5
Cellobiose	+	+	TP	1	L-Cysteine	−	−	TN	2,3,4,5
D-Deoxyribose	−	−	TN	1	L-Glutamic acid	−	+	FP	2,3,4,5
D-Xylose	−	−	TN	1	L-Glutamine	+	+	TP	2
L-Arabinose	−	−	TN	1	L-Glycine	+	+	TP	2,3,4,5
L-Rhammose	−	−	TN	1	L-Histidine	−	−	TN	2,3,4,5
D-Mannose	−	−	TN	1	L-Isoleucine	−	−	TN	2,3,4,5
Esculin	+	−	FN	1	L-Leucine	−	−	TN	2,3,4,5
Salicin	+	−	FN	1	L-Lysine	+	+	TP	2,3,4,5
Glycerol	−	−	TN	1	L-Methionine	−	−	TN	2,3,4,5
D-Mannitol	−	−	TN	1	L-Phenylalanine	−	−	TN	2,3,4,5
L-Sorbitol	−	−	TN	1	L-Proline	+	+	TP	2,3,4,5
L-Malic acid	−	−	TN	1	L-Serine	+	−	FN	2,3,4,5
Citric acid	−	−	TN	1	L-Threonine	−	−	TN	2,3,4,5
Fumaric acid	−	−	TN	1	L-Tryptophan	−	−	TN	2,3,4,5
**Nucleotides**					L-Tyrosine	−	−	TN	2,3,4,5
Adenine	+	+	TP	2	L-Valine	−	−	TN	2,3,4,5
Guanine	+	+	TP	2	**Vitamins**				
Xanthine	+	+	TP	2	4-Aminobenzoic acid	+	+	TP	2
Cytosine	+	+	TP	2	Biotin	+	+	TP	2
Thymine	+	+	TP	2	Choline	+	+	TP	2
Uracil	+	+	TP	2	Cyanocobalamin	+	+	TP	2
**Minerals**					Folic acid	+	+	TP	2
MnSO_4_ · 4 H_2_O	−	−	TN	2	Nicotinic acid	−	−	TN	2
MgSO_4_ · 7 H_2_O	+	+	TP	2	D-Pantothenate	−	−	TN	2
K_2_HPO_4_	−	−	TN	2	Pyridoxine	+	+	TP	2
CaCl_2_	+	+	TP	2	Riboflavin	+	+	TP	2
CuSO_4_ · 5 H_2_O	+	+	TP	2	Thiamine	+	+	TP	2
FeSO_4_ · 7 H_2_O	+	+	TP	2					
ZnSO_4_ · 7 H_2_O	+	+	TP	2					

*1, Beelman et al. (1977), 2, Terrade and Mira de Orduña (2009), 3, Garvie (1967), 4, Fourcassie et al. (1992), 5, Remize et al. (2006)*.

*We compared 61 in vivo experiments with in silico simulations under different media conditions. From the 61 experiments, we obtained 30 true positives (TP), 27 true negatives (TN), 1 false positive (FP), and 3 false negative (FN). + growth is achieved by O. oeni; − growth is not achieved by O. oeni*.

The *in silico* analysis of nutritional requirements showed that at least one carbon source is required for growth. Thus, one out of the 9 following carbon sources could be employed to sustain growth: glucose, fructose, ribose, galactose, arabinose, cellobiose, trehalose, melibiose, or gluconate. Additionally, the model predicted that 14 amino acids are essential for growth (arginine, cysteine, histidine, isoleucine, methionine, phenylalanine, tryptophan, tyrosine, valine, leucine, threonine, serine, glutamate, and asparagine), and that the 6 remaining ones (alanine, aspartic acid, glutamine, glycine, lysine, and proline) were not.

Single nucleotide omission experiments of iSM454 showed that these metabolites were not essential for *O. oeni*. On the other hand, nicotinic acid and pantothenate were predicted to be essential nutrients. Moreover, several vitamins, such as biotin, folic acid, pyridoxine, riboflavin, and thiamin were not essential.

The model was able to identify that *O. oeni* was able to grow in 91% of the cases in which growth has been observed experimentally (sensitivity); whereas it identified correctly 96% of the cases where *O. oeni* did not grow (specificity). Furthermore, 97% of the experiments in which *O. oeni* was predicted to grow, *O. oeni* actually grew (precision). Additionally, 90% of the experiments in which *O. oeni* was not predicted to grow, *O. oeni* actually did not grow (NPV). The accuracy of the model was 93%, i.e., the proportion of correct results to total predictions. By comparison, the GEM model of *L. plantarum* presents an accuracy of 86% (Teusink et al., 2005); and the iIN800 model of *S. cerevisiae*, 89% (Nookaew et al., 2008). Finally, the F-score, a measure of the accuracy that can be interpreted as a weighted average of the sensitivity and the precision, was 94%, indicating that overall the model has a very good performance.

We obtained one false positive related to prediction of growth in the absence of L-glutamate, because of the presence of transamination reactions in the network, which artificially allowed the production of this amino acid. On the other hand, we obtained three false negatives related to growth in the absence of L-serine, and growth with esculin and salicin as sole carbon sources.

### Applications

The reconstructed GEM can be employed to study metabolic fluxes, as well as to identify gene or nutrient essentiality *in silico*. In the following, we exemplify some potential uses of the iSM454 model.

#### Prediction of non-growth associated maintenance (NGAM)

In order to predict the NGAM values for each experimental data set, we optimized the growth rate considering a range of possible NGAM values. The value of NGAM that allowed the minimal error at each specific growth rate prediction was selected, reaching an average error in the biomass formation of 0.14% in the two conditions analyzed. These NGAM values accounted for 0.07 and 2.3 mmol of ATP gDW^−1^ h^−1^ at 0 and 12% ethanol, respectively. Thus, when exposed to 12% ethanol, *O. oeni* PSU-1 spends 30 times more ATP to maintain the cellular machinery than in the absence of ethanol.

#### Impact of ethanol concentration on the redistribution of intracellular fluxes

**Figure 5:**
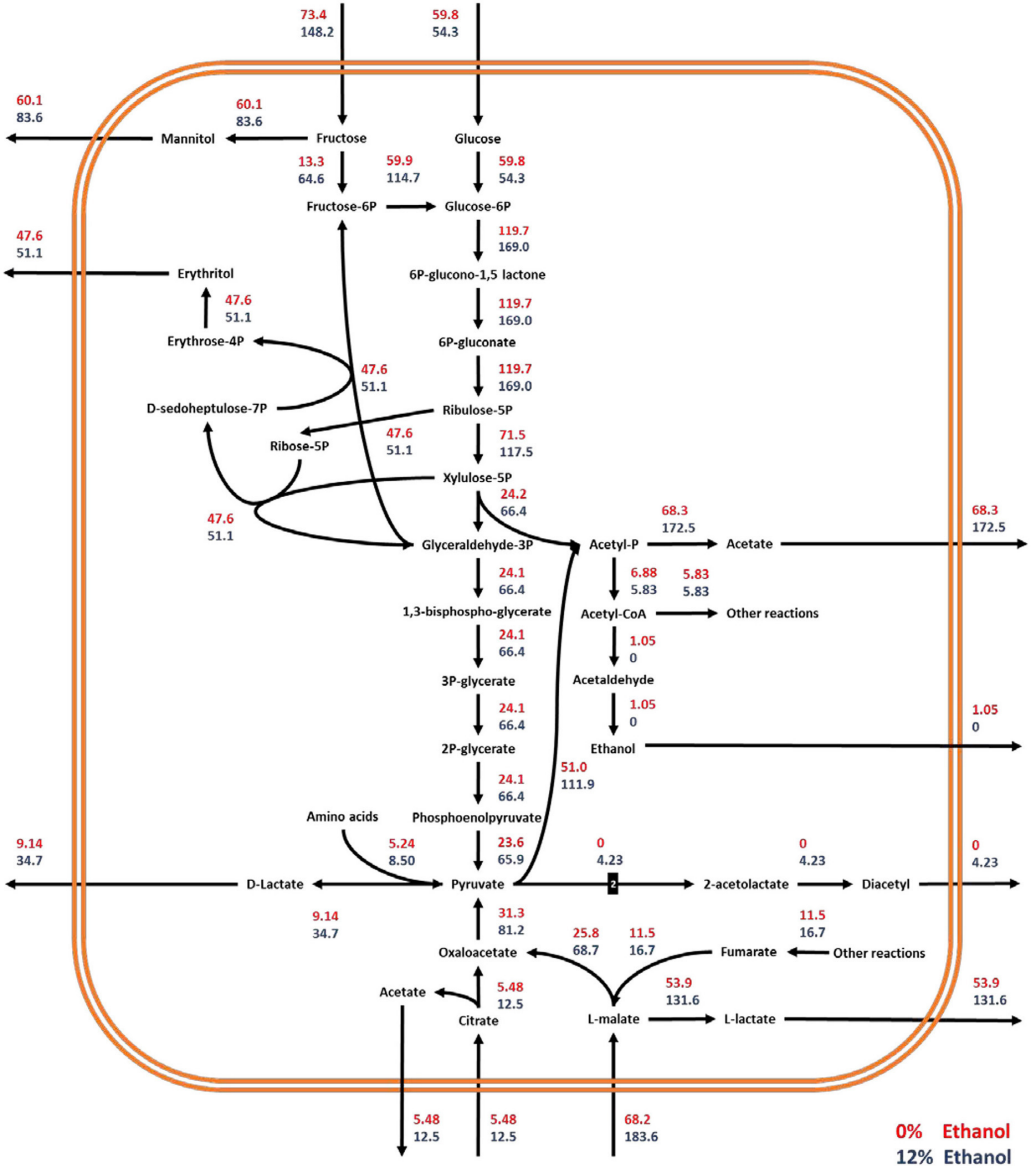
**Metabolic flux redistribution of the central carbon metabolic pathways of *O. oeni* PSU-1 upon cultivation in a culture medium with 0% (red), and 12% (blue) ethanol concentration**. The number “2” in the reaction pyruvate to 2-acetolactate means that 2 moles of pyruvate generate 1 mole of 2-acetolactate.

FBA of experimental data showed that a significant redistribution of intracellular fluxes occurs in the cell when *O. oeni* is grown in the absence of ethanol or under 12% ethanol content. To compare these two conditions, fluxes were standardized by growth rate. The glucose uptake rate is similar for 0 and 12% ethanol (Figure 5). On the contrary, significant changes occur in the consumption rates of fructose, malate, and citrate. The uptake rate of these compounds increases 102, 169, and 127%, respectively, when the bacterium is cultivated with 12%v/v ethanol. The net result is an increase in the fluxes through the heterolactic pathway. Consequently, a higher production rate of D-lactate (279%), L-lactate (144%), acetate (150%), mannitol (39%), and erythritol (7%), was achieved.

Despite the fructose uptake rate more than double in cultures with 12% ethanol, the mannitol production rate only increased by 39%. Indeed, in the absence of ethanol, *Y*_*mannitol*/*fructose*_ was 0.82; meanwhile, at 12% ethanol, this yield decreased to 0.56. Thus, fructose in cultures with ethanol is preferentially transformed to fructose-6-phosphate—and then to glucose-6-phosphate—compared to those without ethanol, which subsequently leads to a higher production of D-lactate and acetate. In fact, considering total carbon source as the sum of glucose, fructose, citrate, and L-malate, resulting *Y*_*D*−*lactate*/*total C*_ and *Y*_*acetate*/*total C*_ were 0.044 and 0.35 in the absence of ethanol; and 0.086 and 0.46 at 12% ethanol, respectively.

The flux through the malolactic reaction was also much faster in ethanol-containing cultures. The uptake rate of L-malate increased 169%, and the concomitant production rate of L-lactate, 144%. It is worthy to note that in both cases, not all the L-malate was transformed to L-lactate. A minor fraction is transformed to oxaloacetate through the malate dehydrogenase. Interestingly, *Y*_*L*−*lactate*/*L*−*malate*_ slightly decreased from 0.79 to 0.71, suggesting that for cells grown at 12% ethanol, a higher part of L-malate is destined to oxaloacetate.

Ethanol content significantly impacts the production rate of diacetyl, which increases from 0 to 4.23 mmol gDW^−1^ h^−1^. Regarding erythritol, even though its production remains almost the same in both conditions *Y*_*erythritol*/*glucose*+*fructose*_ decreased from 0.36 to 0.25, suggesting that a higher extent of fructose and glucose is transformed into other metabolites than erythritol.

As expected from the higher carbon flow through the heterolactic pathway in ethanol-containing cultures, the corresponding ATP specific production rate was 3-fold faster than in the absence of ethanol, passing from 0.74 to 1.98 mmol gDW^−1^ h^−1^ for 0 and 12% ethanol, respectively. These were calculated by adding the fluxes of acetate kinase (EC 2.7.2.1), pyruvate kinase (EC 2.7.1.40), and phosphoglycerate kinase (EC 2.7.2.3); and subtracting the fluxes through hexokinase (EC 2.7.1.1/E.C 2.7.1.2) and fructokinase (EC 2.7.1.4). On the other hand, by using the usual method for ATP determination through heterolactic fermentation, which consists of adding the total D-lactate and acetate produced, the resulting ATP production rates were 1.38 and 2.12 mmol gDW^−1^ h^−1^, for 0 and 12% ethanol-containing cultures, respectively.

**Figure 6:**
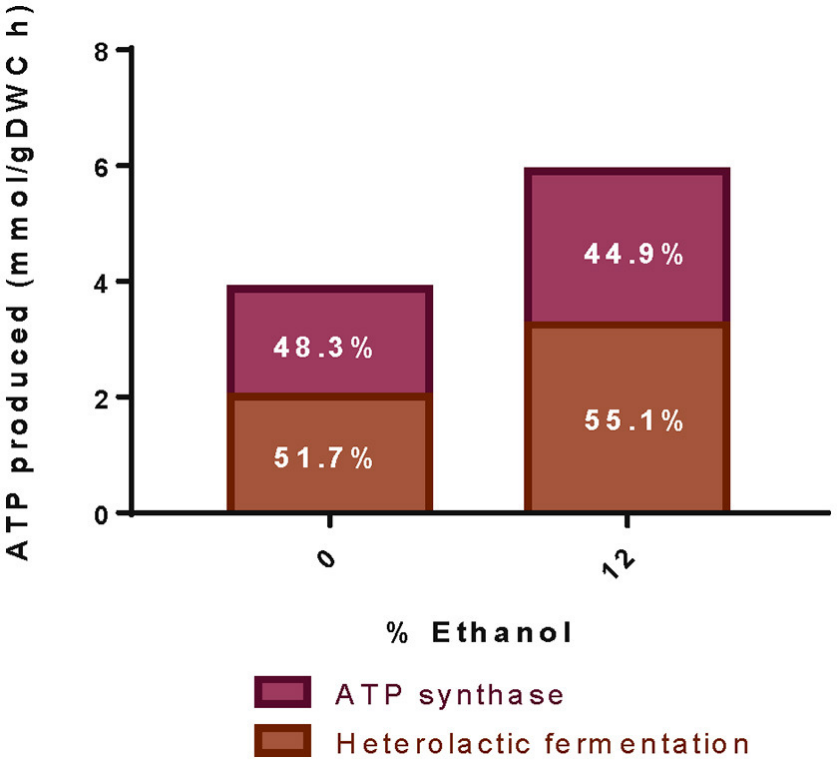
**Production of ATP at different ethanol concentrations**. The figure illustrates the total ATP produced during simulation of experimental data at pH 4.8 and at different ethanol concentrations. Numbers indicate the ATP production either by heterolactic fermentation (dark bars) or ATP Synthase (light bars).

Total ATP production increases as the concentration of ethanol in the medium increases (Figure 6). The model includes ATP generation by both, heterolactic fermentation and ATP synthase. At high ethanol content, the percentage of ATP produced via ATP synthase slightly decreases, from 48 to 45%; while the ATP formed through the heterolactic fermentation increases, correspondingly.

#### Sensitivity analysis

We assessed the impact of exchange reactions on the growth of *O. oeni*, by conducting a sensitivity analysis (Table 5) through estimation of the reduced costs, as well as of the scaled reduced costs, associated with these constrained fluxes. This methodology has been used before to assess the impact of metabolic reactions on ATP formation in the lactic acid bacterium *Lactobacillus plantarum* (Teusink et al., 2006). Reduced costs allow quantifying how much the objective flux could be improved by changing a capacity constraint. Scaled reduced costs represent the reduced cost normalized by the current biomass flux and the flux associated with the constraint. They allow computing the relative effect of a change in a parameter to the whole system.

**Table 5:** **Sensitivity Analysis of the model using experimental data at pH 4.8**.

**Reaction**	**Direction**	**Reduced cost**	**Scaled reduced cost**
β-D-fructose [ex]	Consumed	0.023 ± 0.007	2.585 ± 0.824
α-D-glucose [ex]	Consumed	0.031 ± 0.007	1.670 ± 0.433
D-mannitol [ex]	Produced	−0.035 ± 0.008	−2.671 ± 0.817
Citrate [ex]	Consumed	0.005 ± 0.008	0.033 ± 0.066
(R)-lactate [ex]	Produced	0.003 ± 0.005	0.089 ± 0.154
(S)-lactate [ex]	Produced	0.003 ± 0.005	0.338 ± 0.586
(S)-malate [ex]	Consumed	0.009 ± 0.005	0.860 ± 0.502
L-Cys [ex]	Consumed	0.012 ± 0.003	0.054 ± 0.013
L-Ser [ex]	Consumed	0.005 ± 0.005	0.011 ± 0.012
L-Thr [ex]	Consumed	0.022 ± 0.002	0.057 ± 0.009
Ethanol [ex]	Produced	−0.005 ± 0.009	−0.005 ± 0.009
Acetate [ex]	Produced	0.003 ± 0.002	0.468 ± 0.482
D-erythritol [ex]	Produced	−0.032 ± 0.010	−1.488 ± 0.412

*Reactions analyzed correspond to exchange reactions of the model. Reduced cost quantifies how much the objective flux (specific growth rate) improves by changing a capacity constraint, and scaled reduced cost is the reduced cost normalized by the current biomass flux and the flux associated to the constraint. Positive value indicates an improvement in the specific growth rate, and negative value indicates a decrease*.

The increase of sugar uptake rate—fructose or glucose—had a positive effect on the specific growth rate (scaled reduced costs of 2.59 and 1.67, respectively) as well as the transport of acids -malate or citrate uptake- (scaled reduced costs of 0.86 and 0.03, respectively), and the export of acetate, L- and D-lactate (0.47, 0.34, 0.09 respectively). The specific uptake rate of cysteine, serine and threonine also showed a positive effect on the specific growth rate (scaled reduced costs of 0.05, 0.01, and 0.06, respectively). On the contrary, the production rate of D-mannitol and D-erythritol had a negative effect on the specific growth rate, which showed scaled reduced cost of −2.67 and −1.48, respectively.

#### Exploring the solution space

To evaluate the robustness of our results, we employed random sampling and FVA to identify and explore the different phenotypes achieved at optimal specific growth rate.

**Figure 7:**
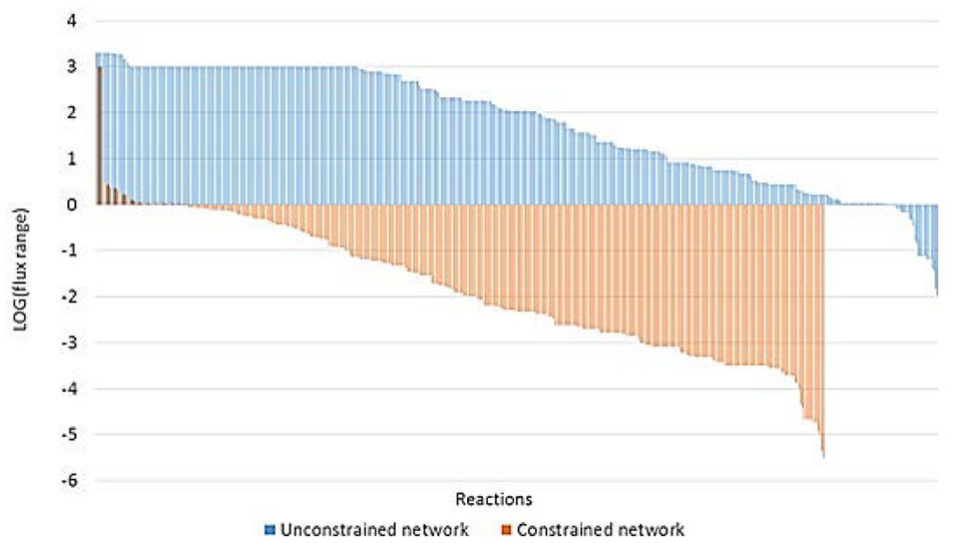
**Flux Variability Analysis for unconstrained and constrained networks**. The figure illustrates the flux range in mmol gDW^−1^ h^−1^ for every non-blocked reaction of the unconstrained (blue) and constrained (orange) network expressed as base 10 logarithm. Flux range of the constrained network was calculated as the average flux ranges of networks applying constraints for 0 and 12% of ethanol content.

FVA revealed some important differences between unconstrained and constrained networks (Figure 7). 485 and 419 reactions were able to carry flux in the unconstrained and constrained network, respectively. The larger differences were found for reactions with a narrow flux range (less than 1 mmol gDW^−1^ h^−1^, i.e., a negative value for the logarithm of the flux range in Figure 7). We found only 30 reactions with a flux range of less than 1 mmol gDW^−1^ h^−1^ in the unconstrained network; meanwhile, this value increased to 370 in the constrained network. Moreover, 99% of the reactions in the constrained network showed a flux range lower than 3 mmol gDW^−1^ h^−1^, suggesting that the constraints applied (uptake and production rates) strongly delimit the solution space. Thus, applying these constraints, the phenotype is well defined.

Additionally, random sampling in the constrained network revealed that the solution space was tight and alternative pathways were limited. Except in the case of reactions of isomer interconversions which could result in a futile cycle, none of the reaction rates analyzed changed more than 1.1 mmol gDW^−1^ h^−1^.

Interestingly, when constrained by experimental rates, FVA shows that *O. oeni* requires oxygen to achieve growth at all ethanol levels. Moreover, the oxygen consumption rates needed for growth increase as the concentration of ethanol increases, ranging from 0.8–1.2 to 4.1–4.2 mmol gDW^−1^ h^−1^ for 0 and 12% ethanol, respectively.

#### *In silico* reaction deletion analysis

The essential reactions of *O. oeni* were predicted by *in silico* simulations of reaction knockouts—inhibiting the activity of the enzyme(s) carrying away the respective reaction. This was conducted by further constraining the model, i.e., fixing the flux of the corresponding reaction to zero.

**Figure 8:**
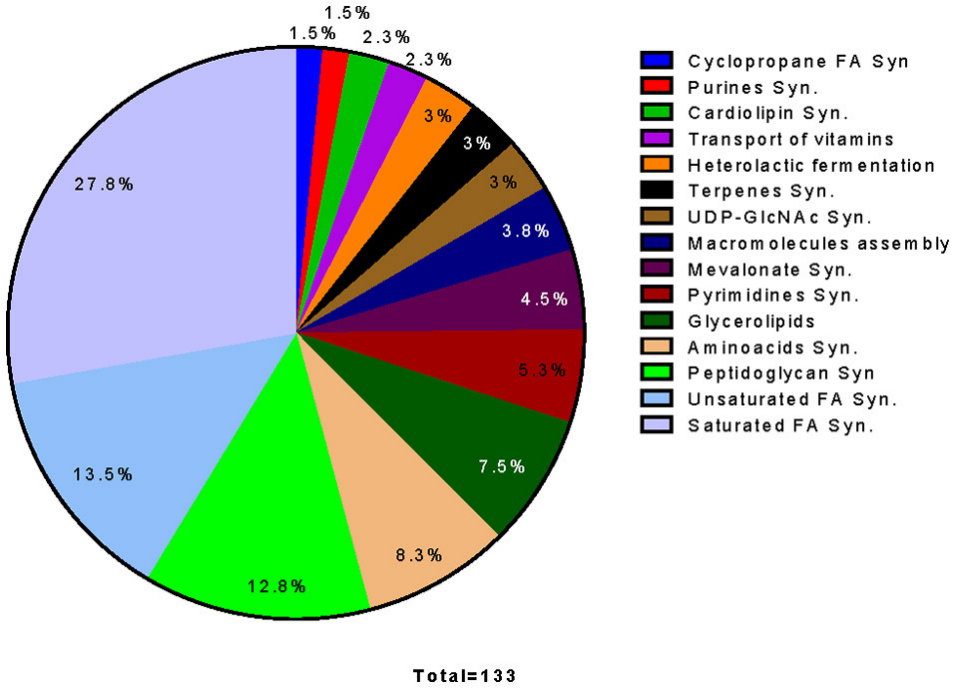
**Pathway distribution of 133 out of 164 essential reactions determined by Reaction Deletion on an *in silico* simulation**. Reactions were classified as essential if growth was affected by at least 80%. Reactions corresponding to non-classified reactions, exchange reactions or pathways that have only one reaction as essential were not considered.

132 essential reactions were found by reaction deletion analysis, which represent 20% of the 660 total reactions of the model; of these, 28% correspond to fatty acid biosynthesis, and 14% to unsaturated fatty acid biosynthesis. The other main essential pathways include the biosynthesis of peptidoglycan (10%), glycerolipids (8%), and amino acid biosynthesis (8%) (Figure 8).

## Discussion

In this work, we reconstructed, curated and validated the first genome-scale metabolic model of *O. oeni* PSU-1. The resulting iSM454 comprises 660 reactions, 536 metabolites and 454 genes, and is able to predict growth under different culture conditions with 93% accuracy. An accurate prediction depends largely on the refinement process. The draft reconstruction was thoroughly curated pathway by pathway, evaluating the stoichiometry, direction, and reversibility of each reaction. After an exhaustive literature search, several pathways were modified in the draft reconstruction, while others were completed, removed, or added to the iSM454 model. Several metabolisms (fatty acids, exopolysaccharides, amino acids, and energetic) were thoroughly curated in the model.

Regarding biosynthesis of unsaturated and cyclopropane fatty acids, we included the reactions for the biosynthesis of palmitoleate (C16:1 Δ9), cis-vaccenic acid (C18:1 Δ11), lactobacillic acid (C19:0 cyΔ11), and dihydrosterculic acid (C19:0 cyΔ9). Moreover, we discovered that the reactions for generating cyclopropane bounds were associated with *OEOE_1176*, a gene related to a generic reaction of cyclization of unsaturated fatty acids. We identified dihydrosterculic acid, which derives from oleic acid under stress conditions (Tracey and Britz, 1989a; Lonvaud-Funel and Desens, 1990; Guerrini et al., 2002), as an essential nutrient in the model. Therefore, an equation for the transport of oleic acid was included.

EPS biosynthesis has been related to the survival of *O. oeni* under stress conditions, such as those prevailing in wine during malolactic fermentation (MLF). Moreover, these polysaccharides have been implicated in ropiness, a wine spoilage process (Dimopoulou et al., 2012). Therefore, we included in the model the detailed biosynthesis of EPS based on the pathways described by Dimopoulou et al. (2012, 2014), which consist of two main products: heteropolysaccharide (glucosyl-rhamnosyl-galactoside) and homopolysaccharide (dextran).

Moreover, the iSM454 model was able to correctly predict most essential amino acids, in agreement with the analysis of amino acids pathways reported by Mills et al. (2005). The model predicts that 14 amino acids are essential and that *O. oeni* is only able to synthesize 6 amino acids, which is supported by previous evidence (Garvie, 1967; Fourcassie et al., 1992; Remize et al., 2006; Terrade and Mira de Orduña, 2009). Mills et al. (2005) found that the biosynthesis pathway of cysteine was complete, which agrees with our reconstruction. However, cysteine has been experimentally found as an essential amino acid in most cases (Garvie, 1967; Fourcassie et al., 1992; Remize et al., 2006; Terrade and Mira de Orduña, 2009). Hence, although its biosynthesis pathway is complete, missing pathways for sulfur assimilation could explain its essentiality for *O. oeni* cells.

Another important feature of the model is the representation of different proton extrusion/energy generation pathways employed by *O. oeni*, particularly the transformation of malic into lactic acid, using the malolactic enzyme (malate decarboxylase). Likewise, a lactic acid transporter that allows this compound to cross the cytoplasmic membrane was added. Citrate metabolism, another important system of energy generation in *O. oeni*, was also included. Both processes allow proton extrusion, which relates to ATP synthase, allowing more ATP to be synthesized. These three added metabolic processes were critical for accurate model performance, especially for the prediction of specific growth rates. Thus, simulations strongly suggest that proton transport is the most important process for the survival of *O. oeni* under these harsh cultural conditions.

We found that accurate proton balancing and cofactor utilization was fundamental for the successful prediction of *O. oeni* phenotype by iSM455 model. For this purpose, we ensure that the only reactions for extrusion of protons were those related to heterolactic fermentation (efflux of lactate and carbon dioxide); in those cases, the network properly described the proton motive force needed to generate energy from the ATP synthase located in the cell membrane. Additionally, a careful revision of the metabolism was performed to account for proper cofactor utilization. This feature was recently reported as a critical step in improving phenotypic predictions by genome-scale metabolic models (Pereira et al., 2016). Therefore, we manually curated all reactions by forcing the use of NADPH/NADP+ in anabolic reactions, e.g., fatty acid biosynthesis, and NADH/NAD+ for catabolic reactions.

The total ATP production rate calculated by the model was 26% less, on average, than the ATP production rate estimated using the approach of other authors (Salou et al., 1994; Zhang and Lovitt, 2005, 2006). This difference could result from several reasons: (i) pyruvate, the precursor of D-lactate, can be produced either from oxaloacetate, malate or even from some amino acids, such as cysteine, serine, or threonine or consumed for the synthesis of diacetyl and ethanol; (ii) acetate can be directly produced from citrate or acetaldehyde, not yielding any ATP; and (iii) production of erythritol requires that some carbon leaves the heterolactic pathway to generate its precursor, erythrose 4 phosphate; therefore, the ATP consumed by the hexokinase could not be regenerated downstream. Mink et al. (2015) showed that diacetyl production could be induced by exogenous pyruvate; the authors concluded that any substrate increasing intracellular pyruvate could induce the synthesis of diacetyl, as observed in a manner consistent with our results.

In general, microorganisms colonizing extreme ecological niches need higher energetic requirements for cell maintenance, which is not reflected in biomass formation (Russell and Cook, 1995). Therefore, a relevant applied use of iSM454 was the determination of m-ATP, which represents non-growth associated maintenance (NGAM) requirements. Using our experimental data, the model was able to predict m-ATP of 0.07 and 2.3 mmol gDW^−1^ h^−1^ for *O. oeni* PSU-1 grown under 0 and 12% v/v ethanol, respectively. These m-ATP values allowed predicting biomass with an average error of 0.14%. Despite the remarkable importance of m-ATP for visualization of cell behavior under stress conditions, there is scarce information in the literature about this parameter for *O. oeni*. Zhang and Lovitt (2006) determined an NGAM of 0.6 mmol ATP g DW^−1^ h^−1^ for *O. oeni* 11,648 strain, when growing in continuous culture at pH 4.5 with glucose and fructose as carbon sources and in the absence of ethanol. We determined that the stress produced by 12% ethanol in the medium required a 30-fold increment in the m-ATP needed, compared to the cultivation without ethanol. Interestingly, growth associated maintenance (GAM) was on average 0.25 mmol ATP gDW^−1^, pointing out that under ethanol stress (12%v/v), *O. oeni* spends in total almost nine times more energy in cell maintenance than under non-stress conditions, i.e., absence of ethanol in the medium.

To the best of our knowledge, this is the first report determining the ATP required for maintaining cells of *O. oeni* growing in a medium with ethanol. It is worthy to note that model predictions were performed using experimental data obtained in a wine-simulated environment, where this microorganism commonly develops. As a consequence, the model can effectively be used to predict internal fluxes in cases where there is scarce growth. This is relevant for winemakers because many strains are able to perform MLF in spite of achieving scarce growth in wine. The model is particularly useful in this case because it predicts MLF, whereas other mathematical models of *O. oeni* cannot (Fahimi et al., 2014; Brandam et al., 2016). Furthermore, the model is able to predict internal metabolic fluxes, determining the production of mannitol, erythritol, acetate, diacetyl, acetoin, among other compounds that could have significant organoleptic impacts on the resulting wine.

As expected for the effect of variations in the constrained fluxes, the sensitivity analysis showed positive reduced costs associated with the uptake rate of D-glucose, D-fructose, and D-malic acid, as well as an increase in the production of DL-lactate and acetate. It is expected that a higher rate for these reactions leads to a higher specific growth rate because more ATP can be obtained directly through heterolactic—from D-glucose and D-fructose—and indirectly through malolactic fermentation—from D-malic acid. Interestingly, the uptake of D-fructose has higher scaled reduced cost, meaning that it has the greatest effect on growth rate, probably because of the importance of the regeneration of redox factors. Zhang and Lovitt (2005) reported that the specific growth rate of *O. oeni* NCIMB 11648 increased when the ratio among glucose and fructose concentrations in the medium was reduced from 0.5 to 0.3, supporting our results related to the impact on the specific growth rate of increasing fructose uptake rate. On the other hand, an increase in the uptake rate of citric acid and some amino acids such as L-cysteine, L-serine and L-threonine also leads to a higher growth rate, in accordance with the essentiality of these amino acids. All these metabolites have been identified as important contributors to energy metabolism in lactic acid bacteria (Teusink et al., 2006), which support their role in the specific growth rate.

Sensitivity analyses demonstrated that an increase in the production of D-mannitol or D-erythritol has a negative effect on growth rate. At first sight, this could be counterintuitive. Nevertheless, *O. oeni* needs reduced redox cofactors for anabolic processes and biomass formation, which are used during the biosynthesis of mannitol and/or erythritol.

The iSM454 model allowed finding a direct relationship between several metabolic fluxes and ethanol content in the medium. Fructose and amino acid consumption rates increased concomitantly with ethanol content. Moreover, erythritol, D-lactate and acetate production rates also increased in ethanol-containing cultures. As expected, the metabolic fluxes related with malic acid consumption and L-lactic production rates increased with ethanol. These compounds are critical for regeneration of redox cofactors such as NADH/NAD+, which restore the redox balance inside the cell. Our results therefore confirm previous studies that point to redox balancing as a survival strategy for *O. oeni* (Salou et al., 1994; Maicas et al., 1999; Zhang and Lovitt, 2005).

Moreover, a redistribution of intracellular fluxes occurred when ethanol content increased in the culture medium. FVA revealed that *O. oeni* requires oxygen to grow at all ethanol levels. Furthermore, oxygen requirements increase concomitantly with ethanol concentration. Although the calculated oxygen specific consumption rates were low, they had a similar value (6.25 mmol gDW^−1^ h^−1^) to those determined by Aceituno et al. (2012) for the wine yeast strain *Saccharomyces cerevisiae* EC1118, grown in nitrogen–limited continuous cultures, sparged with 1.2 μM of oxygen. These results confirm the microaerophilic behavior of *O. oeni*. Oxygen is mainly used for pyruvate oxidation by pyruvate oxidase (E.C.1.2.3.3), threonine degradation by aminoacetone:oxygen oxidoreductase (E.C. 1.4.3.21) and spontaneous diacetyl formation by acetoin oxidation.

The overall good performance of the metabolic model evidences the correct distribution of metabolic fluxes, with or without 12% ethanol content and pH 4.8. However, it is worthy to mention that the information about *O. oeni* is still scarce; the physiological as well as proteomic and transcriptomic responses of the bacterium grown under different environmental perturbations are necessary to further improve the model. These might include determination of biomass composition, exhaustive electronic and proton balancing of stoichiometric equations and integration of transcriptomic and proteomic data. On the other hand, dynamic flux balance analysis (dFBA) has emerged as a promising strategy to study batch cultures of several strains (Sainz et al., 2003; Hanly et al., 2012; Sánchez et al., 2014). Indeed, this methodology has been already applied to understand the behavior of industrial *Saccharomyces cerevisiae* strains in wine-like medium (Vargas et al., 2011). Therefore, a dFBA for *O. oeni* could be useful to simulate the kinetics of growth and industrial MLF extension.

Finally, a future challenge is the development of a more extended platform, based on the iSM454 model, allowing the simulation and prediction of the biological interactions occurring within the wine microbiome. For example, *E. coli's* GEM has been successfully employed as a platform to model metabolite exchange between different organism under different environmental conditions (Jain and Srivastava, 2009; Klitgord and Segre, 2010; Wintermute and Silver, 2010). The consortium metabolic models could also be applied to winemaking to simulate and understand the interactions between *O. oeni* and other microorganisms that share this ecological niche, as *S. cerevisiae* and other LAB, like *Lb. plantarum, Lb. kunkeii, Pediococcus pentosaceus*; and even undesirable and detrimental wine microorganisms, like *Brettanomyces spp*. or *Acetobacter aceti*, responsible for acetic acid production spoilage. Consortium metabolic models might predict how each organism develops in a shared, not isolated, scenario (Tzamali et al., 2011). The iSM454 model would be a valuable tool to be employed for further modeling *O. oeni* in coexistence with other species.

## Author contributions

EA designed the research and provided guide throughout the investigation. EA, SM, and PC coordinated the project. SM and PC are first-authorships and developed and refined the model, and PC developed the OMIX version of the reconstruction. SM and MR performed the model validation and evaluation. AC designed, performed, and coordinated the batch experiments as well as processed the HPLC data. All the authors wrote the paper, read, and approved the final manuscript.

### Conflict of interest statement

The authors declare that the research was conducted in the absence of any commercial or financial relationships that could be construed as a potential conflict of interest.

## Footnotes

^1^http://www.genome.jp/kegg/

^2^https://metacyc.org/

^3^http://www.membranetransport.org/transportDB2/index.html

^4^http://blog.theseed.org/servers/

^5^https://blast.ncbi.nlm.nih.gov/Blast.cgi

^6^http://www.gurobi.com/company/news/highlights-of-gurobi-optimizer-6.5

## Abbreviations

ATP: Adenosine triphosphate
dFBA: dynamic Flux Balance Analysis
EPS: Exopolysaccharide
FBA: Flux Balance Analysis
FN: False Negative
FP: False Positive
FVA: Flux Variability Analysis
GAM: Growth Associated Maintenance
GEM: Genome-scale Model
GENRE: Genome-scale Reconstruction
LAB: Lactic Acid Bacteria
LP: Linear programming
MLF: Malolactic Fermentation
NGAM: Non-Growth Associated Maintenance
TN: True Negative
TP: True Positive
PPP: Pentose Phosphate Pathway.

## References

[B1] Aceituno F. F. Orellana M. Torres J. Mendoza S. Slater A. W. Melo F.. (2012). Oxygen response of the wine yeast *Saccharomyces cerevisiae* EC1118 grown under Carbon-Sufficient, nitrogen-limited enological conditions. Appl. Environ. Microbiol. 78, 8340–8352. 10.1128/AEM.02305-12 23001663 PMC3497381

[B2] Aung H. W. Henry S. A. Walker L. P. (2013). Revising the representation of fatty acid, glycerolipid, and glycerophospholipid metabolism in the consensus model of yeast metabolism. Ind. Biotechnol. 9, 215–228. 10.1089/ind.2013.0013 24678285 PMC3963290

[B3] Bartowsky E. J. (2005). *Oenococcus oeni* and malolactic fermentation–moving into the molecular arena. Aust. J. Grape Wine Res. 11, 174–187. 10.1111/j.1755-0238.2005.tb00286.x

[B4] Bartowsky E. J. Costello P. J. Chambers P. J. (2015). Emerging trends in the application of malolactic fermentation. Aust. J. Grape Wine Res. 21, 663–669. 10.1111/ajgw.12185

[B5] Bartowsky E. J. Francis I. L. Bellon J. R. Henschke P. A. (2002). Is buttery aroma perception in wines predictable from the diacetyl concentration? Aust. J. Grape Wine Res. 8, 180–185. 10.1111/j.1755-0238.2002.tb00254.x

[B6] Bauer R. Dicks L. (2004). Control of malolactic fermentation in wine. A review. South Afr. J. Enol. Vitic. 25, 74–88. Available online at: http://hdl.handle.net/10019.1/78805

[B7] Beelman R. B. Iii A. G. Keen R. M. (1977). A new strain of Leuconostoc oenos for induced malo-lactic fermentation in eastern wines. Am. J. Enol. Vitic. 28, 159–165.

[B8] Borneman A. R. McCarthy J. M. Chambers P. J. Bartowsky E. J. (2012). Comparative analysis of the *Oenococcus oeni* pan genome reveals genetic diversity in industrially-relevant pathways. BMC Genomics 13:373. 10.1186/1471-2164-13-373 22863143 PMC3472311

[B9] Brandam C. Fahimi N. Taillandier P. (2016). Mixed cultures of *Oenococcus oeni* strains: a mathematical model to test interaction on malolactic fermentation in winemaking. LWT - Food Sci. Technol. 69, 211–216. 10.1016/j.lwt.2016.01.045

[B10] Campbell-Sills H. El Khoury M. Favier M. Romano A. Biasioli F. Spano G.. (2015). Phylogenomic analysis of *Oenococcus oeni* reveals specific domestication of strains to cider and wines. Genome Biol. Evol. 7, 1506–18. 10.1093/gbe/evv084 25977455 PMC4494047

[B11] Caspi R. Altman T. Billington R. Dreher K. Foerster H. Fulcher C. A.. (2014). The MetaCyc database of metabolic pathways and enzymes and the BioCyc collection of pathway/genome databases. Nucleic Acids Res. 42, 459–471. 10.1093/nar/gkt1103 24225315 PMC3964957

[B12] Ciezack G. Hazo L. Chambat G. Heyraud A. Lonvaud-Funel A. Dols-Lafargue M. (2010). Evidence for exopolysaccharide production by *Oenococcus oeni* strains isolated from non-ropy wines. J. Appl. Microbiol. 108, 499–509. 10.1111/j.1365-2672.2009.04449.x 19659698

[B13] Costantini A. Rantsiou K. Majumder A. Jacobsen S. Pessione E. Svensson B.. (2015). Complementing DIGE proteomics and DNA subarray analyses to shed light on *Oenococcus oeni* adaptation to ethanol in wine-simulated conditions. J. Proteomics 123, 114–127. 10.1016/j.jprot.2015.04.019 25920369

[B14] Dale J. M. Popescu L. Karp P. D. (2010). Machine learning methods for metabolic pathway prediction. BMC Bioinformatics 11:15. 10.1186/1471-2105-11-15 20064214 PMC3146072

[B15] Davis C. R. Wibowo D. Eschenbruch R. Lee T. H. Fleet G. H. (1985). Practical implications of malolactic fermentation: a review. Am. J. Enol. Vitic. 36, 290–301.

[B16] De Man J. C. Rogosa M. Sharpe M. E. (1960). A medium for the cultivation of Lactobacilli. J. Appl. Bacteriol. 23, 130–135. 10.1111/j.1365-2672.1960.tb00188.x

[B17] Dimopoulou M. Hazo L. Dols-Lafargue M. (2012). Exploration of phenomena contributing to the diversity of *Oenococcus oeni* exopolysaccharides. Int. J. Food Microbiol. 153, 114–22. 10.1016/j.ijfoodmicro.2011.10.024 22119266

[B18] Dimopoulou M. Vuillemin M. Campbell-Sills H. Lucas P. M. Ballestra P. Miot-Sertier C.. (2014). Exopolysaccharide (EPS) synthesis by *Oenococcus oeni*: from genes to phenotypes. PLoS ONE 9:e98898. 10.1371/journal.pone.0098898 24901216 PMC4047060

[B19] Droste P. Miebach S. Niedenführ S. Wiechert W. Nöh K. (2011). Visualizing multi-omics data in metabolic networks with the software Omix: a case study. Biosystems 105, 154–61. 10.1016/j.biosystems.2011.04.003 21575673

[B20] Fahimi N. Brandam C. Taillandier P. (2014). A mathematical model of the link between growth and L-malic acid consumption for five strains of *Oenococcus oeni*. World J. Microbiol. Biotechnol. 30, 3163–3172. 10.1007/s11274-014-1743-8 25248866

[B21] Feist A. M. Herrgard M. J. Thiele I. Reed J. L. Palsson B. Ø. (2009). Reconstruction of biochemical networks in microbial organisms. Nat. Rev. Microbiol. 7, 129–143. 10.1038/nrmicro1949 19116616 PMC3119670

[B22] Flahaut N. A. L. Wiersma A. van de Bunt B. Martens D. E. Schaap P. J. Sijtsma L.. (2013). Genome-scale metabolic model for *Lactococcus lactis* MG1363 and its application to the analysis of flavor formation. Appl. Microbiol. Biotechnol. 97, 8729–39. 10.1007/s00253-013-5140-2 23974365

[B23] Fourcassie P. Belarbi A. Maujean A. (1992). Growth, D-glucose utilization and malolactic fermentation by Leuconostoc œnos strains in 18 media deficient in one amino acid. J. Appl. Bacteriol. 73, 489–496. 10.1111/j.1365-2672.1992.tb05010.x

[B24] Garbay S. Rozes N. Lonvaud-Funel A. (1995). Fatty acid composition of Leuconostoc oenos, incidence of growth conditions and relationship with malolactic efficiency. Food Microbiol. 12, 387–395. 10.1016/S0740-0020(95)80120-0

[B25] Garvie E. I. (1967). The growth factor and amino acid requirements of species of the genus Leuconostoc, including Leuconostoc paramesenteroides (sp. nov.) and *Leuconostoc oenos*. J. Gen. Microbiol. 48, 439–447. 10.1099/00221287-48-3-439 6052634

[B26] Gockowiak H. Henschke P. A. (2003). Interaction of pH, ethanol concentration and wine matrix on induction of malolactic fermentation with commercial “direct inoculation” starter cultures. Aust. J. Grape Wine Res. 9, 200–209. 10.1111/j.1755-0238.2003.tb00271.x

[B27] Gurobi Optimization Inc (2016). Gurobi Optimizer Reference Manual. Houston, TX: Gurobi Optimization, Inc.

[B28] Guerrini S. Bastianini A. Granchi L. Vincenzini M. (2002). Effect of oleic acid on *Oenococcus oeni* strains and malolactic fermentation in wine. Curr. Microbiol. 44, 5–9. 10.1007/s00284-001-0066-9 11727034

[B29] Hanly T. J. Urello M. Henson M. A. (2012). Dynamic flux balance modeling of *S. cerevisiae* and *E. coli* co-cultures for efficient consumption of glucose/xylose mixtures. Appl. Microbiol. Biotechnol. 93, 2529–2541. 10.1007/s00253-011-3628-1 22005741

[B30] Henschke P. A. (1993). An overview of malolactic fermentation research. Aust. Zeal. Wine Ind. J. 8, 69–79.

[B31] Jain R. Srivastava R. (2009). Metabolic investigation of host/pathogen interaction using MS2-infected *Escherichia coli*. BMC Syst. Biol. 3:121. 10.1186/1752-0509-3-121 20042079 PMC2813233

[B32] Kanehisa M. (2000). Post-genome Informatics. New York, NY: Oxford University Press

[B33] Karp P. D. Paley S. Romero P. (2002). The pathway tools software. Bioinformatics 18, S225–S232. 10.1093/bioinformatics/18.suppl_1.S225 12169551

[B34] Klitgord N. Segre D. (2010). Environments that induce synthetic microbial ecosystems. PLoS Comput. Biol. 6:e1001002. 10.1371/journal.pcbi.1001002 21124952 PMC2987903

[B35] Konings W. N. N. Lolkema J. S. S. Bolhuis H. Van Veen H. W. W. Poolman B. Driessen A. J. M. (1997). The role of transport processes in survival of lactic acid bacteria. Antonie Van Leeuwenhoek 71, 117–128. 10.1023/A:1000143525601 9049023

[B36] Kuepfer L. Sauer U. Blank L. M. (2005). Metabolic functions of duplicate genes in *Saccharomyces cerevisiae*. Genome Res. 15, 1421–1430. 10.1101/gr.3992505 16204195 PMC1240085

[B37] Kunkee R. E. (1974). Malo-Lactic Fermentation and Winemaking, in Chemistry of Winemaking Advances in Chemistry Series, ed Webb A. D. (Washington, DC: American Chemical Society), 151–170.

[B38] Le Marrec C. Bon E. Lonvaud-Funel A. (2007). Tolerance to high osmolality of the lactic acid bacterium *Oenococcus oeni* and identification of potential osmoprotectants. Int. J. Food Microbiol. 115, 335–42. 10.1016/j.ijfoodmicro.2006.12.039 17320992

[B39] Loira N. Dulermo T. Nicaud J.-M. Sherman D. (2012). A genome-scale metabolic model of the lipid-accumulating yeast Yarrowia lipolytica. BMC Syst. Biol. 6:35. 10.1186/1752-0509-6-35 22558935 PMC3443063

[B40] Lonvaud-Funel A. Desens C. (1990). Constitution en acides gras des membranes des bactéries lactiques du vin Incidences des conditions de culture. Sci. Aliments 10, 817–829.

[B41] Madden T. (2002). The BLAST sequence analysis tool, in The NCBI Handbook, eds McEntyre J. Ostell J. (Bethesda, MD: National Center for Biotechnology Information), 1–15.

[B42] Maicas S. González-Cabo P. Ferrer S. Pardo I. (1999). Production of *Oenococcus oeni* biomass to induce malolactic fermentation in wine by control of pH and substrate addition. Biotechnol. Lett. 21, 349–353. 10.1023/A:1005498925733

[B43] Makarova K. S. Slesarev A. Wolf Y. I. Sorokin A. Mirkin B. Koonin E. V.. (2006). Comparative genomics of the lactic acid bacteria. Proc. Natl. Acad. Sci. U.S.A. 103, 15611–6. 10.1073/pnas.0607117103 17030793 PMC1622870

[B44] McCloskey D. Palsson B. Ø. Feist A. M. (2013). Basic and applied uses of genome-scale metabolic network reconstructions of *Escherichia coli*. Mol. Syst. Biol. 9, 661. 10.1038/msb.2013.18 23632383 PMC3658273

[B45] Megchelenbrink W. Huynen M. Marchiori E. (2014). optGpSampler: an improved tool for uniformly sampling the solution-space of genome-scale metabolic networks. PLoS ONE 9:e86587. 10.1371/journal.pone.0086587 24551039 PMC3925089

[B46] Mills D. A. Rawsthorne H. Parker C. Tamir D. Makarova K. S. (2005). Genomic analysis of *Oenococcus oeni* PSU-1 and its relevance to winemaking. FEMS Microbiol. Rev. 29, 465–75. 10.1016/j.femsre.2005.04.011 16125008

[B47] Mink R. Kölling R. Sommer S. Schmarr H. Scharfenberger-schmeer M. (2015). Diacetyl formation by *Oenococcus oeni* during winemaking induced by exogenous pyruvate. Am. J. Enol. Vitic. 66, 85–90. 10.5344/ajev.2014.14056

[B48] Nookaew I. Jewett M. Meechai A. Thammarongtham C. Laoteng K. Cheevadhanarak S.. (2008). The genome-scale metabolic model iIN800 of *Saccharomyces cerevisiae* and its validation: a scaffold to query lipid metabolism. BMC Syst. Biol. 2:71. 10.1186/1752-0509-2-71 18687109 PMC2542360

[B49] Oddone G. M. Mills D. A. Block D. E. (2009). A dynamic, genome-scale flux model of *Lactococcus lactis* to increase specific recombinant protein expression. Metab. Eng. 11, 367–81. 10.1016/j.ymben.2009.07.007 19666133

[B50] Olguín N. Champomier-Vergès M. Anglade P. Baraige F. Cordero-Otero R. Bordons A.. (2015). Transcriptomic and proteomic analysis of *Oenococcus oeni* PSU-1 response to ethanol shock. Food Microbiol. 51, 87–95. 10.1016/j.fm.2015.05.005 26187832

[B51] Oliveira A. P. Nielsen J. Förster J. (2005). Modeling *Lactococcus lactis* using a genome-scale flux model. BMC Microbiol. 5:39. 10.1186/1471-2180-5-39 15982422 PMC1185544

[B52] Orth J. D. Thiele I. Palsson B. Ø. (2010). What is flux balance analysis? Nat. Biotechnol. 28, 245–248. 10.1038/nbt.1614 20212490 PMC3108565

[B53] Park J. M. Kim T. Y. Lee S. Y. (2009). Constraints-based genome-scale metabolic simulation for systems metabolic engineering. Biotechnol. Adv. 27, 979–988. 10.1016/j.biotechadv.2009.05.019 19464354

[B54] Pastink M. I. Teusink B. Hols P. Visser S. De Vos W. M. Hugenholtz J. (2009). Genome-scale model of *Streptococcus thermophilus* LMG18311 for metabolic comparison of lactic acid bacteria. Appl. Environ. Microbiol. 75, 3627–3633. 10.1128/AEM.00138-09 19346354 PMC2687286

[B55] Pereira R. Nielsen J. Rocha I. (2016). Improving the flux distributions simulated with genome-scale metabolic models of *Saccharomyces cerevisiae*. Metab. Eng. Commun. 3, 153–163. 10.1016/j.meteno.2016.05.002 PMC5779720 29468121

[B56] Remize F. Gaudin A. Kong Y. Guzzo J. Alexandre H. Krieger-Weber S.. (2006). *Oenococcus oeni* preference for peptides: qualitative and quantitative analysis of nitrogen assimilation. Arch. Microbiol. 185, 459–469. 10.1007/s00203-006-0116-6 16775752

[B57] Ren Q. Chen K. Paulsen I. T. (2007). TransportDB: a comprehensive database resource for cytoplasmic membrane transport systems and outer membrane channels. Nucleic Acids Res. 35, D274–D279. 10.1093/nar/gkl925 17135193 PMC1747178

[B58] Ribéreau-Gayon P. Dubourdieu D. Donèche B. Lovaud A. (2006). Handbook of Enology Vol. 1, The Microbiology of Wine and Vinifications, 2nd Edn. Chichester: John Wiley & Sons, Ltd.

[B59] Rodionov D. A. Vitreschak A. G. Mironov A. A. Gelfand M. S. (2003). Regulation of lysine biosynthesis and transport genes in bacteria: yet another RNA riboswitch? Nucleic Acids Res. 31, 6748–6757. 10.1093/nar/gkg900 14627808 PMC290268

[B60] Russell J. B. Cook G. M. (1995). Energetics of bacterial growth: balance of anabolic and catabolic reactions. Microbiol. Rev. 59, 48–62. 7708012 10.1128/mr.59.1.48-62.1995 PMC239354

[B61] Sainz J. Pizarro F. Pérez-Correa J. R. Agosin E. (2003). Modeling of yeast metabolism and process dynamics in batch fermentation. Biotechnol. Bioeng. 81, 818–828. 10.1002/bit.10535 12557315

[B62] Salema M. Lolkema J. S. San Romão M. V. Loureiro-Dias M. C. (1996). The proton motive force generated in *Leuconostoc oenos* by L-malate fermentation. J. Bacteriol. 178, 3127–3132. 10.1128/jb.178.11.3127-3132.1996 8655490 PMC178062

[B63] Salou P. Loubser P. Pareilleux A. (1994). Growth and energetics of *Leuconostoc oenos* during cometabolism of glucose with citrate or fructose. Appl. Environ. Microbiol. 60, 1459–66. 8017930 10.1128/aem.60.5.1459-1466.1994 PMC201503

[B64] Sánchez B. J. Pérez-Correa J. R. Agosin E. (2014). Construction of robust dynamic genome-scale metabolic model structures of *Saccharomyces cerevisiae* through iterative re-parameterization. Metab. Eng. 25, 159–173. 10.1016/j.ymben.2014.07.004 25046158

[B65] Smith R. L. (1984). Efficient monte carlo procedures for generating points uniformly distributed over bounded regions. Oper. Res. 32, 1296–1308. 10.1287/opre.32.6.1296

[B66] Terrade N. Mira de Orduña R. (2009). Determination of the essential nutrient requirements of wine-related bacteria from the genera Oenococcus and Lactobacillus. Int. J. Food Microbiol. 133, 8–13. 10.1016/j.ijfoodmicro.2009.03.020 19446351

[B67] Teusink B. van Enckevort F. H. J. Francke C. Wiersma A. Wegkamp A. Smid E. J.. (2005). *In silico* reconstruction of the metabolic pathways of *Lactobacillus plantarum*: comparing predictions of nutrient requirements with those from growth experiments. Appl. Environ. Microbiol. 71, 7253–7262. 10.1128/AEM.71.11.7253-7262.2005 16269766 PMC1287688

[B68] Teusink B. Wiersma A. Molenaar D. Francke C. de Vos W. M. Siezen R. J.. (2006). Analysis of growth of *Lactobacillus plantarum* WCFS1 on a complex medium using a genome-scale metabolic model. J. Biol. Chem. 281, 40041–8. 10.1074/jbc.M606263200 17062565

[B69] Thiele I. Palsson B. Ø. (2010). A protocol for generating a high-quality genome-scale metabolic reconstruction. Nat. Protoc. 5, 93–121. 10.1038/nprot.2009.203 20057383 PMC3125167

[B70] Tracey R. P. Britz T. (1989a). The effect of amino acids on malolactic fermentation by *Leuconostoc oenos*. J. Appl. Bacteriol. 67, 589–595.

[B71] Tracey R. P. Britz T. J. (1989b). Cellular fatty acid composition of *Leuconostoc oenos*. J. Appl. Bacteriol. 66, 445–456. 10.1111/j.1365-2672.1989.tb05114.x

[B72] Tzamali E. Poirazi P. Tollis I. G. (2011). A computational exploration of bacterial metabolic diversity identifying metabolic interactions and growth-efficient strain communities. BMC Syst. Biol. 5:167. 10.1186/1752-0509-5-167 22008379 PMC3212978

[B73] Varela C. A. Agosin E. Baez M. E. Klapa M. Stephanopoulos G. (2003). Metabolic flux redistribution in *Corynebacterium glutamicum* in response to osmotic stress. Appl. Microbiol. Biotechnol. 60, 547–55. 10.1007/s00253-002-1120-7 12536254

[B74] Vargas F. A. Pizarro F. Pérez-Correa J. R. Agosin E. (2011). Expanding a dynamic flux balance model of yeast fermentation to genome-scale. BMC Syst. Biol. 5:75. 10.1186/1752-0509-5-75 21595919 PMC3118138

[B75] Verouden M. P. H. Notebaart R. A. Westerhuis J. A. van der Werf M. J. Teusink B. Smilde A. K. (2009). Multi-way analysis of flux distributions across multiple conditions. J. Chemom. 23, 406–420. 10.1002/cem.1238

[B76] Williams S. A. Hodges R. A. Strike T. L. (1984). Cloning the gene for the malolactic fermentation of wine from *Lactobacillus delbrueckii* in *Escherichia coli* and yeasts. Appl. Environ. Microbiol. 47, 288–293. 16346469 10.1128/aem.47.2.288-293.1984 PMC239661

[B77] Wintermute E. H. Silver P. A. (2010). Emergent cooperation in microbial metabolism. Mol. Syst. Biol. 6, 1–7. 10.1038/msb.2010.66 20823845 PMC2964121

[B78] Zapparoli G. Tosi E. Azzolini M. Vagnoli P. Krieger S. (2009). Bacterial inoculation strategies for the achievement of Malolactic fermentation in high-alcohol wines. South Afr. J. Enol. Vitic. 30, 49–55. 10.21548/30-1-1424

[B79] Zhang D. Lovitt R. (2006). Performance assessment of malolactic fermenting bacteria *Oenococcus oeni* and *Lactobacillus brevis* in continuous culture. Appl. Microbiol. Biotechnol. 69, 658–64. 10.1007/s00253-005-0021-y 16012836

[B80] Zhang D. S. Lovitt R. W. (2005). Studies on growth and metabolism of *Oenococcus oeni* on sugars and sugar mixtures. J. Appl. Microbiol. 99, 565–572. 10.1111/j.1365-2672.2005.02628.x 16108798

