# Supplementary figures and images for: Genome-Scale Reconstruction of the Metabolic Network in Oenococcus oeni to Assess Wine Malolactic Fermentation
Source: Front Microbiol. 2017 Mar 30;8:534. doi: 10.3389/fmicb.2017.00534 (PMC5372704; doi:10.3389/fmicb.2017.00534)

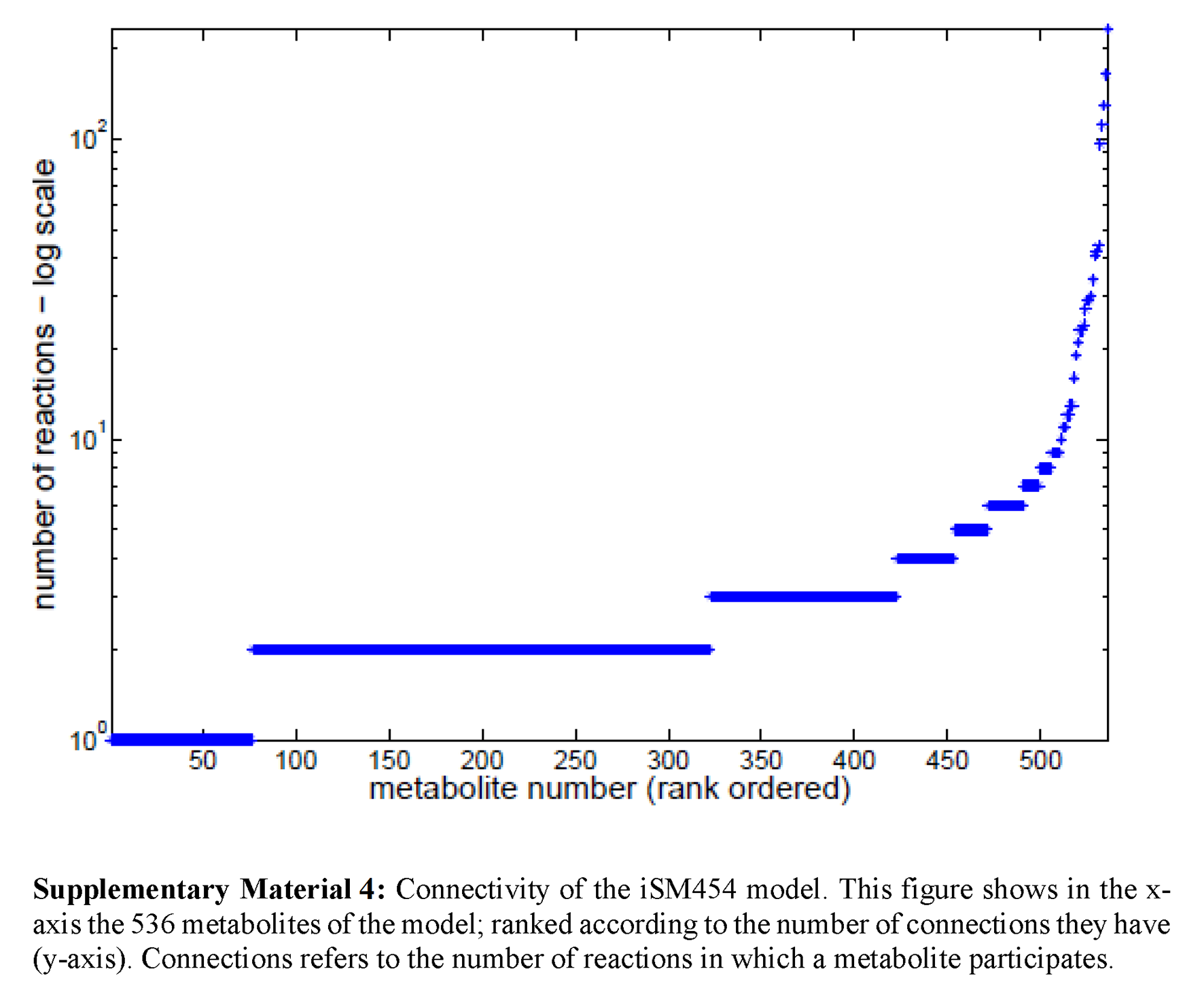

Supplement: Supplementary file 4 [file Image2.tiff]
