# Supplementary material for: Genome-Scale Reconstruction of the Metabolic Network in Oenococcus oeni to Assess Wine Malolactic Fermentation
Source: Front Microbiol. 2017 Mar 30;8:534. doi: 10.3389/fmicb.2017.00534 (PMC5372704; doi:10.3389/fmicb.2017.00534)
Supplement: Supplementary file 3 [file Image1.pdf]

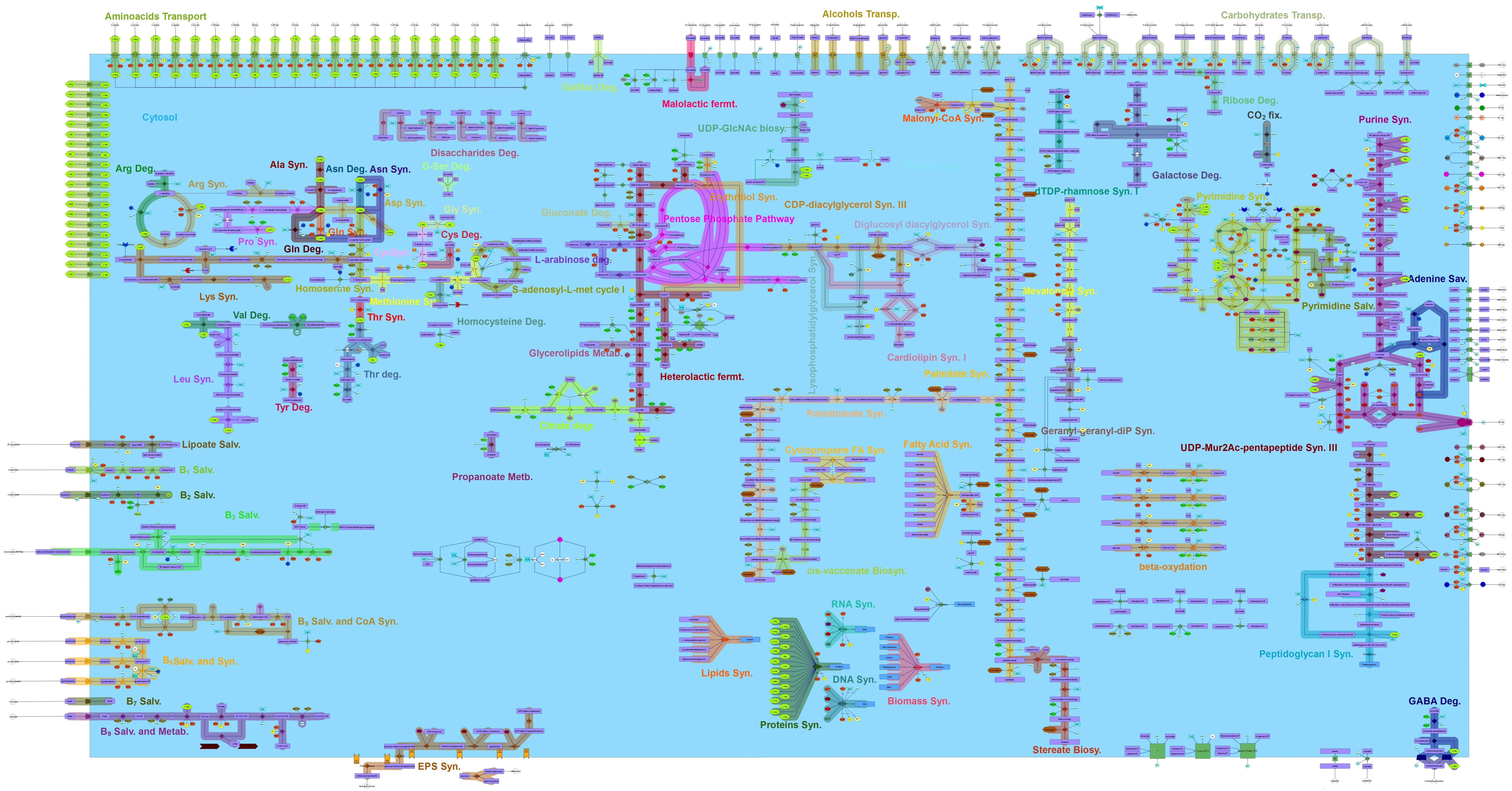

Supplementary Material 3: Genome-Scale metabolic model of *Oenococcus oeni.* The matabolic map was developed using Omix 1.8 (Droste et al., 2011)
